# Phosphoproteomic dysregulation in Huntington’s disease mice is rescued by environmental enrichment

**DOI:** 10.1093/braincomms/fcac305

**Published:** 2022-11-21

**Authors:** Isaline Mees, Shanshan Li, Harvey Tran, Ching-Seng Ang, Nicholas A Williamson, Anthony J Hannan, Thibault Renoir

**Affiliations:** Florey Institute of Neuroscience and Mental Health, Melbourne Brain Centre, University of Melbourne, Parkville, VIC 3010, Australia; Florey Institute of Neuroscience and Mental Health, Melbourne Brain Centre, University of Melbourne, Parkville, VIC 3010, Australia; Florey Institute of Neuroscience and Mental Health, Melbourne Brain Centre, University of Melbourne, Parkville, VIC 3010, Australia; Bio21 Mass Spectrometry and Proteomics Facility, University of Melbourne, Parkville, VIC 3010, Australia; Bio21 Mass Spectrometry and Proteomics Facility, University of Melbourne, Parkville, VIC 3010, Australia; Florey Institute of Neuroscience and Mental Health, Melbourne Brain Centre, University of Melbourne, Parkville, VIC 3010, Australia; Faculty of Medicine, Dentistry and Health Sciences, University of Melbourne, Parkville, VIC 3010, Australia; Florey Institute of Neuroscience and Mental Health, Melbourne Brain Centre, University of Melbourne, Parkville, VIC 3010, Australia; Faculty of Medicine, Dentistry and Health Sciences, University of Melbourne, Parkville, VIC 3010, Australia

**Keywords:** Huntington’s disease, transgenic mice, phosphoproteomics, environmental enrichment

## Abstract

Huntington’s disease is a fatal autosomal-dominant neurodegenerative disorder, characterized by neuronal cell dysfunction and loss, primarily in the striatum, cortex and hippocampus, causing motor, cognitive and psychiatric impairments. Unfortunately, no treatments are yet available to modify the progression of the disease. Recent evidence from Huntington’s disease mouse models suggests that protein phosphorylation (catalysed by kinases and hydrolysed by phosphatases) might be dysregulated, making this major post-translational modification a potential area of interest to find novel therapeutic targets. Furthermore, environmental enrichment, used to model an active lifestyle in preclinical models, has been shown to alleviate Huntington’s disease-related motor and cognitive symptoms. However, the molecular mechanisms leading to these therapeutic effects are still largely unknown. In this study, we applied a phosphoproteomics approach combined with proteomic analyses on brain samples from pre-motor symptomatic R6/1 Huntington’s disease male mice and their wild-type littermates, after being housed either in environmental enrichment conditions, or in standard housing conditions from 4 to 8 weeks of age (*n* = 6 per group). We hypothesized that protein phosphorylation dysregulations occur prior to motor onset in this mouse model, in two highly affected brain regions, the striatum and hippocampus. Furthermore, we hypothesized that these phosphoproteome alterations are rescued by environmental enrichment. When comparing 8-week-old Huntington’s disease mice and wild-type mice in standard housing conditions, our analysis revealed 229 differentially phosphorylated peptides in the striatum, compared with only 15 differentially phosphorylated peptides in the hippocampus (statistical thresholds fold discovery rate 0.05, fold change 1.5). At the same disease stage, minor differences were found in protein levels, with 24 and 22 proteins dysregulated in the striatum and hippocampus, respectively. Notably, we found no differences in striatal protein phosphorylation and protein expression when comparing Huntington’s disease mice and their wild-type littermates in environmentally enriched conditions. In the hippocampus, only four peptides were differentially phosphorylated between the two genotypes under environmentally enriched conditions, and 22 proteins were differentially expressed. Together, our data indicates that protein phosphorylation dysregulations occur in the striatum of Huntington’s disease mice, prior to motor symptoms, and that the kinases and phosphatases leading to these changes in protein phosphorylation might be viable drug targets to consider for this disorder. Furthermore, we show that an early environmental intervention was able to rescue the changes observed in protein expression and phosphorylation in the striatum of Huntington’s disease mice and might underlie the beneficial effects of environmental enrichment, thus identifying novel therapeutic targets.

## Teaser

Mees *et al*. report dysregulated protein phosphorylation in the striatum of a transgenic mouse model of Huntington’s disease, which was rescued by an early environmental intervention. The present study identifies novel therapeutic targets for Huntington’s disease, and sheds light on the molecular mechanisms underlying the beneficial effects of environmental enrichment.

## Graphical Abstract

**Graphical abstract:**
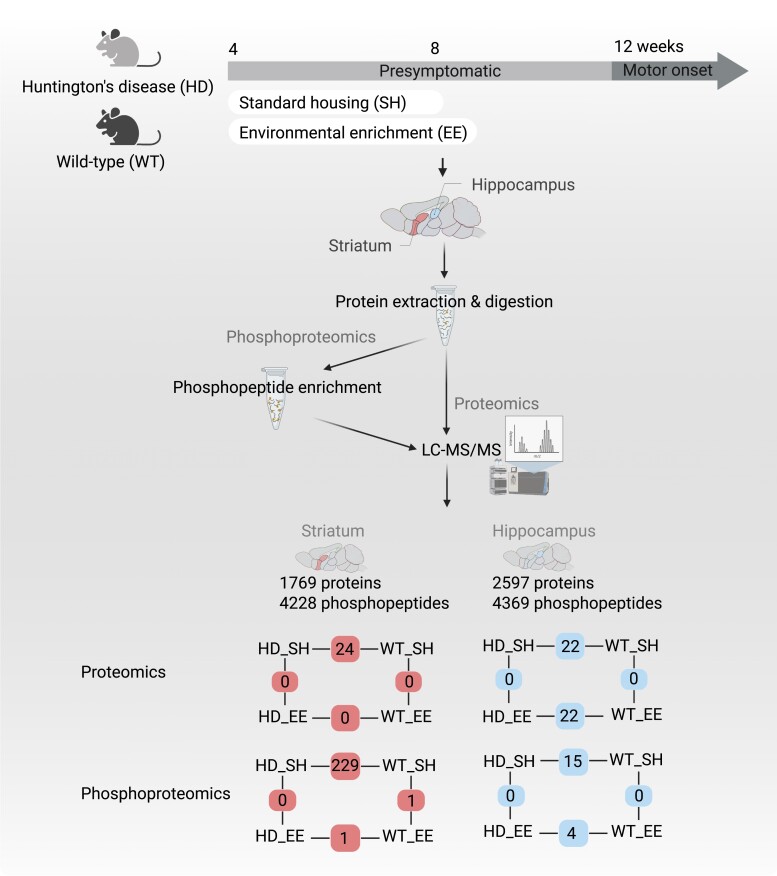

## Introduction

Huntington’s disease is a fatal autosomal-dominant neurodegenerative disorder caused by the expansion of a cytosine-adenine-guanine (CAG) repeat in exon 1 of the huntingtin gene.^1^ The tandem-repeat mutation leads to the formation of a mutant huntingtin protein, which is cleaved, misfolded and forms aggregates in all cells of the body.^2^ Although the huntingtin protein is ubiquitously expressed, the disease is characterized by an exacerbated degeneration of the medium spiny neurons (MSNs) in the striatum, leading to a movement disorder often referred to as ‘Huntington’s chorea’.^3^ Other brain regions are also affected in Huntington’s disease, primarily the cortex and hippocampus, and dysfunction of associated neural circuits may lead to cognitive and psychiatric impairments.^4^ Unfortunately, there is currently no treatment available that can alter the course of the disease, highlighting the need to identify new therapeutic targets.

Transgenic R6/1 mice have been widely used to model Huntington’s disease in preclinical research, recapitulating the disease symptoms, including motor, cognitive and affective impairments.^5–7^ We have recently reported a protein phosphorylation dysregulation in the cortex of R6/1 Huntington’s disease male mice in standard-housing conditions, occurring primarily prior to motor-symptom onset.^8^ Our findings were supported by previously published evidence that protein phosphorylation is dysregulated in other Huntington’s disease mouse models.^9–12^ Protein phosphorylation, catalysed by kinases and hydrolysed by phosphatases, plays a crucial role in cellular signalling, making this molecular process a promising target for the development of new Huntington’s disease treatments. Here, we investigated whether protein phosphorylation dysregulation also occurs in other brain regions affected in Huntington’s disease (i.e. the striatum and hippocampus) and whether this can be modulated by environmental factors, via experimental manipulations of the housing conditions.

Indeed, previous studies have shown that the phenotype of Huntington’s disease mice can be ameliorated in enriched environment conditions.^13–15^ More specifically, EE [when compared with standard housing (SH) conditions] has been shown to delay the onset of striatal-dependent motor impairments^13^ and the onset of hippocampal-dependent long-term memory and spatial learning deficits.^14^ But despite these successful interventions, there is still no clear molecular mechanism of action.

In the present study, we therefore hypothesized that protein phosphorylation dysregulations occur in the striatum and hippocampus of pre-symptomatic Huntington’s disease mice compared with their wild-type (WT) littermates. Moreover, we hypothesized that the beneficial effects of EE drive changes in protein phosphorylation in these key brain regions. We aimed to use the acquired results to identify new drug targets for this devastating disorder.

A proteome analysis was conducted, to verify that the changes in phosphorylation observed were not due to protein expression changes. Here, we report the first proteomics study on R6/1 Huntington’s disease mice, identifying novel proteins differentially expressed (DE) prior to motor symptom onset in this mouse model of Huntington’s disease.

## Materials and methods

### Animals and tissue collection

R6/1 (Huntington’s disease) transgenic hemizygous males were originally obtained from the Jackson Laboratory (Bar Harbor, ME, USA) and bred with CBB6 (CBA∼C57/B6) F1 females to establish the R6/1 colony at the Florey Institute of Neuroscience and Mental Health. After weaning, Huntington’s disease male mice and their WT littermates were group housed (four mice per cage) with two of each genotype.

**Figure 1:**
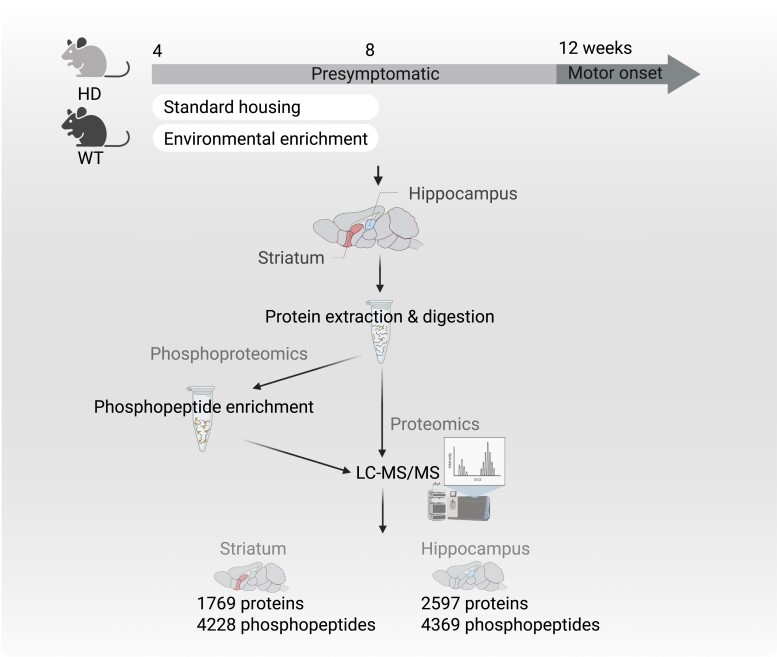
**Experimental design.** Hippocampus and striatum samples were collected from Huntington’s disease mice and their wild-type littermates (WT) at 8 weeks of age; and processed for proteomics and phosphoproteomics analyses. In the striatum, 1769 proteins and 4228 phosphopeptides were quantified. In the hippocampus, 2597 proteins and 4369 phosphopeptides were quantified. WT, wild-type; LC-MS/MS, liquid chromatography with tandem mass spectrometry.

As previously described,^16^ SH cages were 31 × 10 × 16 cm in size, with a layer of sawdust and tissues as nesting material. The environmental enrichment (EE) cages were larger (38 × 28 × 15 cm) and contained more nesting material, shredded paper, a running wheel, and a set of different objects (ladder, shelter, tunnel and random objects). The objects were changed once a week. Mice were randomly assigned to one of these environments from 4 weeks of age to 8 weeks of age (*n* = 6 per group, 24 animals in total) (Fig. 1). Mice were maintained in a 12 h light/dark cycle with access to food and water ad libitum. Mice were culled at 8 weeks of age via cervical dislocation for tissue dissection. Striatum and hippocampus samples were snap-frozen in dry ice before being stored at −80°C. Sample preparation time was minimized to maintain phosphorylation. All experiments were approved and performed in accordance with the guidelines of the Florey Institute Animal Ethics Committee and the National Health and Medical Research Council.

### Sample preparation and phosphopeptide enrichment

Striatum and hippocampus samples from 8-week-old male mice (*n* = 6 per group) were used for protein extraction with a RIPA buffer containing protease and phosphatase inhibitors (PhosSTOP, Roche). Protein concentration in each sample was determined from a BCA assay. We precipitated 500µg of proteins overnight at −20°C, using 5× volume ice cold acetone. The following day, the pellet was solubilized in 8 M Urea in 50 mM TEAB and incubated for 30 min at 37°C. The samples were then treated with 10 mM TCEP and 55 mM IAA. Thereafter, the samples were diluted to 1 M urea with 25 mM TEAB and digested overnight at 37°C with Trypsin/LysC (1:50 protein:enzyme). The next day, the samples were acidified to 1% (v/v) formic acid. We used Oasis HLB 60 mg cartridges for solid-phase extraction. The cartridges were first washed with 80% ACN containing 0.1% TFA, followed by another washing with 0.1% TFA. The samples were then loaded onto the cartridges and washed with 0.1% TFA. The proteins were eluted with 80% ACN containing 0.1% TFA. A final BCA assay was performed to allow equal loading across all samples. Twenty milligrams of protein from each sample were kept for the proteomics experiment and resuspended in a buffer containing 2% ACN, 0.05% TFA. The remaining samples were then freeze-dried until phospho-enrichment. All samples were prepared in two separate batches, with samples from the four experimental groups present in each batch, and were randomized before LC-MS/MS analysis.

For the phosphopeptide enrichment, TiO beads (6:1 TiO:peptides) were washed with 50% ACN, 5% TFA (washing buffer) and incubated for 10 min with 2 M lactic acid in 5% TFA, 50% ACN (loading buffer). The TiO beads in loading buffer were added to the peptides samples and incubated for 1 h. Subsequently, the phosphopeptides were washed and eluted with 1% ammonia followed by elution with 30% ACN. The samples were then acidified with 1 ul of formic acid per 10 ul eluent and freeze-dried. The phosphopeptides were resuspended in a buffer containing 2% ACN, 0.05% TFA just before LC-MS/MS analysis.

### Mass spectrometry analyses

Samples were analysed by nanoESI-LC-MS/MS using an Orbitrap Exploris 480 mass spectrometer (Thermo Scientific) equipped with a nanoflow reversed-phase-HPLC (Ultimate 3000 RSLC, Dionex). The LC system was equipped with an Acclaim Pepmap nano-trap column (Dinoex-C18, 100 Å, 75 µm × 2 cm) and an Acclaim Pepmap RSLC analytical column (Dinoex-C18, 100 Å, 75 µm × 50 cm). The tryptic peptides were injected (concentration of 1µg on column, for the proteomics and phosphoproteomics) to the enrichment column at an isocratic flow of 5 µL/min of 2% v/v CH_3_CN containing 0.1% v/v formic acid for 5 min applied before the enrichment column was switched in-line with the analytical column. The eluents were 5% DMSO in 0.1% v/v formic acid (solvent A) and 5% DMSO in 100% v/v CH_3_CN and 0.1% v/v formic acid (solvent B). The flow gradient was (i) 0–6 min at 3% B, (ii) 6–95 min, 3–22% B, (iii) 95–105 min 22–40% B, (iv) 105–110 min, 40–80% B, (v) 110–115 min, 80–80% B, (vi) 115–117 min, 80–3% and equilibrated at 3% B for 10 min before the next sample injection. All spectra were acquired in positive ionization mode with full scan MS acquired from m/z 300–1600 in the FT mode at a mass resolving power of 120 000, after accumulating to an AGC target value of 3.0E6, with a maximum accumulation time of 25 ms. The RunStart EASY-IC lock internal lockmass was used. Data-dependent HCD MS/MS of charge states > 1 was performed using a 3 s scan method, at a normalized AGC target of 100%, automatic injection, a normalized collision energy of 30%, and spectra acquired at a resolving power of 15 000. Dynamic exclusion was used for 20 s.

Raw files were processed using the MaxQuant proteomics software package (version 2.0.1.0) with the Andromeda search engine for protein and peptide identification.^17^ The results were searched against a *Mus Musculus* database (SwissProt, Taxonomy ID 10090, downloaded April 2021) and using the default search parameters. Trypsin was selected as the cleavage enzyme, cysteine carbamidomethyl was selected as fixed modification and methionine oxidation, serine, threonine and tyrosine phosphorylation as variable modifications. The match between run option was selected. Protein and peptides groups were set to a maximum false discovery rate (FDR) of < 0.01. Each raw file was considered as one experiment.

### Bioinformatics and statistical analyses

#### Phosphoproteomics analysis

The processed data was analysed with Perseus (version 1.6.14.0). First, we removed contaminants and reverse peptides from the matrix. Peptides with a phosphate localization probability higher than 0.75 were kept for further analysis. After expanding the site table, phosphopeptides intensities were log2 transformed and samples were annotated with their condition (i.e. WT_SH, WT_EE, HD_SH and HD_EE). Phosphoproteome characterization is available in supplementary Figure 1. Phosphopeptides with valid values in 100% of the samples in at least one group were kept for statistical analysis. Following this, we normalized the intensities of the phosphopeptides in each sample by subtracting the median and imputed missing values from normal distribution (0.3 width, 1.8 down shift). We then verified that the imputed missing values necessary for the statistical analysis were indeed low in samples where a specific phosphopeptide was not detected and did not skew the data in anyway. In addition, we also defined the list of phosphopeptides that are present or absent in all four replicates (Supplementary Tables 1 and 2). For the striatum phosphoproteomics dataset, samples 32 (HD_EE, low detection) and 69 (WT_SH, outlier) were discarded. For the hippocampus phosphoproteomics, sample 68 was discarded from the analysis (HD_SH, low detection). In the striatum, 4228 phosphopeptides were detected with enough valid values (100% of valid values in at least one group) to allow differential expression analysis. In hippocampus samples, 4369 phosphopeptides were retained for differential expression analysis.

#### Phosphoproteomics statistical analysis

The reproducibility of the experiment was assessed with Pearson’s correlation between biological replicates in all groups. The mean Pearson’s correlation coefficient for striatum samples (*r*) was 0.9407 for WT_SH (*n* = 5), 0.9272 for HD_SH (*n* = 6), 0.9332 for WT_EE (*n* = 6) and 0.9107 for HD_EE (*n* = 5). For hippocampus samples, we report a mean Pearson’s correlation of 0.948 for WT_SH (*n* = 6), 0.929 for HD_SH (*n* = 5), 0.951 for WT_EE (*n* = 6) and 0.927 for HD_EE (*n* = 6).

Significant changes in the phosphoproteome between genotypes or housing conditions were defined by Student’s *t*-tests, truncated by permutation-based FDR significance threshold of 0.05 with 250 randomizations, and 1.5 absolute fold change (FC).

#### Proteomics and statistical analysis

The processed data was analysed with Perseus (version 1.6.14.0). First, we removed contaminants, reverse, and only identified by site peptides from the matrix. The data was then log2 transformed and filtered by valid values, with a minimum of four valid values in each group. The intensities of peptides in each sample were normalized by subtracting the median and missing values were imputed from normal distribution (0.3 width, 1.8 down shift). We then verified that the imputed missing values necessary for the statistical analysis were indeed low in samples and did not skew the data in anyway. In addition, we also defined a list of proteins that are present or absent in all four replicates (Supplementary Tables 3 and 4).

The reproducibility of the experiment was assessed with Pearson’s correlation (*r*), which was high between samples within each group in the striatum (WT_SH: 0.9763, WT_EE: 0.9759, HD_SH: 0.9779, HD_EE: 0.9783, *n* = 6 per group) and hippocampus (WT_SH: 0.9933, WT_EE: 0.9935, HD_SH: 0.9938, HD_EE: 0.9924, *n* = 6 per group). In the striatum, 1769 proteins had at least four valid measurements in each group and were retained for statistical analysis. In the hippocampus samples, 2597 proteins were retained for further analysis. Significant changes in the proteome between genotypes or housing conditions were defined by Student's *t*-tests, truncated by permutation-based FDR significance threshold of 0.05% with 250 randomizations, and 1.5 absolute FC.

### Gene ontology and pathway analysis

The DE and phosphorylated proteins were entered into Panther (Pantherdb.org) and IPA (Qiagen) to retrieve protein class, canonical pathways and upstream regulators.

## Results

### Extensive dysregulation of the phosphoproteome in the striatum of Huntington’s disease mice in standard housing conditions

**Figure 2:**
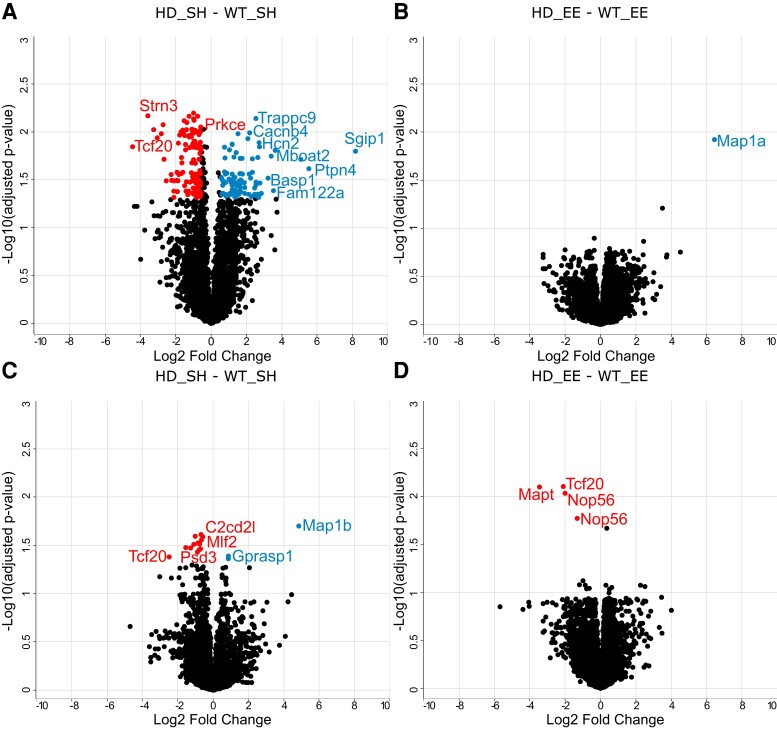
**Protein phosphorylation changes in the striatum and hippocampus of Huntington’s disease mice under standard housing versus environmental enrichment conditions.** Volcano plots of statistical significance against fold change, highlighting the significant phosphopeptides in the striatum between HD_SH and WT_SH (**A**) and between HD_EE and WT_EE (**B**); and in the hippocampus between HD_SH and WT_SH (**C**) and between HD_EE and WT_EE (**D**). Each dot represents a phosphorylation site. For example, the two different Nop56 datapoints on panel (**D**) represent two different phosphorylation sites of the same protein. Significant phosphopeptides (FDR 5%, FC 1.5) are highlighted in colour: left of the zero on the x-axis (red) are downregulated in Huntington’s disease, right of the zero on the x-axis (blue) are upregulated in Huntington’s disease. WT, wild-type; SH, standard housing; EE, environmental enrichment.

When comparing Huntington’s disease and WT mice in SH conditions, we reported 229 DE phosphopeptides in the striatum (Fig. 2A). Of these, 101 phosphopeptides (83 phosphoproteins) were upregulated, and 128 (98 phosphoproteins) were downregulated in Huntington’s disease compared with WT mice. In proteomics, due to the stochastic nature of sampling by the mass spectrometer and/or detection limits, non-identification does not necessarily mean complete absence of a particular protein. However, a phosphopeptide being present/absent in all four replicates could suggest that phosphopeptide is indeed significant. These phosphopeptides are presented in Supplementary Table 1. We found that nine of the proteins whose phosphorylation was downregulated in Huntington’s disease compared with WT mice were also downregulated at the protein level (Supplementary Figure 2). For this reason, these nine phosphoproteins were excluded from the dataset for Panther and IPA analyses.

The Panther analysis revealed that the most prevalent protein classes for the differentially phosphorylated proteins between Huntington’s disease and WT mice in SH included transporters (15 proteins) and protein-modifying enzymes (15 proteins). The transporter class included multiple subunits of voltage-gated ion channels, including ones permeable for calcium (CACNA1C, E; CACNB2,4; CACNG2,4, CBARP), sodium (SCN2A) and potassium (KCND2 and KCNQ2). Several non-receptor serine/threonine protein kinases were also found differentially phosphorylated in the striatum of Huntington’s disease mice (Table 1). We found a high number of differentially phosphorylated cytoskeletal and scaffold proteins (13 proteins each). The cytoskeletal proteins mainly involved actin and actin-binding proteins (SYNPO, ACTL6A, AFDN and ADD1,2,3). For some phosphosites, including the top 10 upregulated and downregulated (Table 2), as well as the ones mapping to kinases (Table 1), we mention if their upstream regulation and downstream functions are known (from phosphosite.org database).

**Table 1:** Differentially phosphorylated kinases in the striatum of Huntington’s disease compared with WT mice in standard housing conditions

Protein symbol	Protein name	Phosphorylation in Huntington’s disease	Residue	PhosphositePlus
CAMK2A; CAMK2B; CAMK2D; CAMK2G	Calcium/calmodulin-dependent protein kinase type II	↓	S234	—
PRKCE	Protein kinase C epsilon type	↓	S337	—
MAP3K9	Mitogen-activated protein kinase kinase kinase 9	↓	S622	—
SPEG	Striated muscle-specific serine/threonine-protein kinase	↓	S493	—
SPEG	Striated muscle-specific serine/threonine-protein kinase	↓	S2361	—
MAP3K10	Mitogen-activated protein kinase kinase kinase 10	↓	S583	—
RPS6KA5	Ribosomal protein S6 kinase alpha-5	↓	S862	—
CAMKK2	Calcium/calmodulin-dependent protein kinase kinase 2	↓	S495	Phosphorylation by cAMP protein kinase A (PKA): enzymatic inhibition, impairment of calcium-calmodulin activation^19^
MINK1	Misshapen-like kinase 1	↓	S644	—
CAMK2A	Calcium/calmodulin-dependent protein kinase type II subunit alpha	↓	T337	Blocks calcium-calmodulin binding, dephosphorylated by PTEN^20^
MARK2	Serine/threonine-protein kinase MARK2	↑	S483	—
SPEG	Striated muscle-specific serine/threonine-protein kinase	↑	S2135	—
PI4KB	Phosphatidylinositol 4-kinase beta	↑	S511	Phosphorylated by PKA, induces localization of enzyme in nucleus^21^
RAF1	Proto-oncogene serine/threonine-protein kinase	↑	S621	Autophosphorylation at Ser621 necessary to avoid proteasome degradation^22^
BAZ1B	Tyrosine-protein kinase	↑	S1464	—
BAZ1B	Tyrosine-protein kinase	↑	S1468	—

**Table 2:** Top 10 up- and downregulated phosphorylation residues in the striatum of Huntington’s disease compared with WT mice

Protein symbol	Protein name	Residue	−log(*P*-value)	log2(FC)	Biological process	PhosphoSitePlus
TCF20	Transcription factor 20	S612	3.4725	−4.4360	DNA-binding transcription factor activity	—
STRN3	Striatin-3	S257	4.8191	−3.5701	Calmodulin binding, negative regulation of transcription	—
JPH4	Junctophilin-4	T172	4.5153	−3.2611	Cross-talk between cell surface and intracellular calcium release	—
ATG4B	Cysteine protease ATG4B	S383	3.8025	−3.0545	Endopeptidase activity, autophagy	Phosphorylated by MST4, induced enzymatic activity and autophagic flux^23^
TCF20	Transcription factor 20	S588	3.8777	−2.8260	DNA-binding transcription factor activity	—
PSD	PH and SEC7 domain-containing protein 1	S156	5.8585	−2.7082	Guanine nucleotide exchange factor, cytoskeleton remodelling	—
CACNB1	Voltage-dependent L-type calcium channel subunit beta-1	T418	3.1459	−2.6574	Calcium ion transport	—
FAM171B	Protein FAM171B	S752	2.6290	−2.5435	Unknown	—
SYNPO	Synaptopodin	S882	2.1970	−2.1103	Actin binding, cytoskeleton organization	—
CRKL	Crk-like protein	S107	2.3650	−2.1083	Activates Ras and Jun kinases signalling pathway	—
HCN2	Potassium/sodium hyperpolarization-activated cyclic nucleotide-gated channel 2	S795	3.5025	2.7518	Cellular response to cAMP, ion transmembrane transport	—
	Uncharacterized protein C1orf21 homologue	S115	2.5783	2.7687	Unknown	—
SRRM2	Serine/arginine repetitive matrix protein 2	S1339	2.3098	2.8553	mRNA splicing	—
BASP1	Brain acid soluble protein 1	S131	2.6986	3.2284	Negative regulation of transcription	—
EIF3B	Eukaryotic translation initiation factor 3 subunit B	S68	6.7151	3.3981	Translation initiation, protein synthesis	—
FAM122A	Protein FAM122A	S34	2.3730	3.5166	Inhibitor of PP2A activity	Phosphorylated by CHK1, induces activity of PP2A^24^
MBOAT2	Lysophospholipid acyltransferase 2	S332	3.2901	3.6223	Lipid modification	—
HCN2	Potassium/sodium hyperpolarization-activated cyclic nucleotide-gated channel 2	S795	3.0927	5.0681	Cellular response to cAMP, ion transmembrane transport	—
PTPN4	Tyrosine-protein phosphatase non-receptor type 4	S899	2.9403	5.5266	Tyrosine phosphatase activity	—
SGIP1	SH3-containing GRB2-like protein 3-interacting protein 1	S491	8.3419	8.1776	Clathrin-mediated endocytosis	—

Top differentially phosphorylated peptides in HD_SH compared with WT_SH in the striatum, sorted by log2FC, with description of the biological processes in which the phosphorylated protein is involved and if available, the function of the specific phosphosites (retrieved on phosphosite.org).

FC, fold change; negative/positive Log2(FC) indicates the phosphorylated residue is downregulated/upregulated in Huntington’s disease mice; − indicates no known functional annotation or upstream regulator.

**Figure 3:**
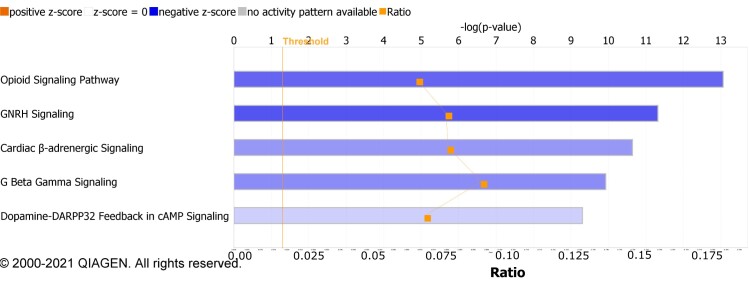
Top 5 canonical pathways in IPA for the differentially phosphorylated proteins in the striatum between WT and Huntington’s disease, in standard-housing conditions.

Pathway analysis from IPA revealed that the Opioid Signalling Pathway (*P*-value 7.94E-14) was the most significant pathway for the changes occurring in the striatal phosphoproteome of Huntington’s disease mice prior to motor symptoms (Fig. 3). The analysis reported a negative *Z*-score (−1.213) for the Opioid Signalling Pathway, indicating an inactivation of this pathway in the striatum of Huntington’s disease mice, likely due to changes in the phosphorylation profiles of the proteins involved (ADCY5, ARRB1, CACNA1C; E; B1; B2; B4; G2; G4, Calm1, CAMK2A, GNAO1, GNG12, GRIN2A, ITPR1, PRKCB, PRKCE, RAF1, RASD2, RGS8 and RGS9, RPS6KA5, TH).

**Figure 4:**
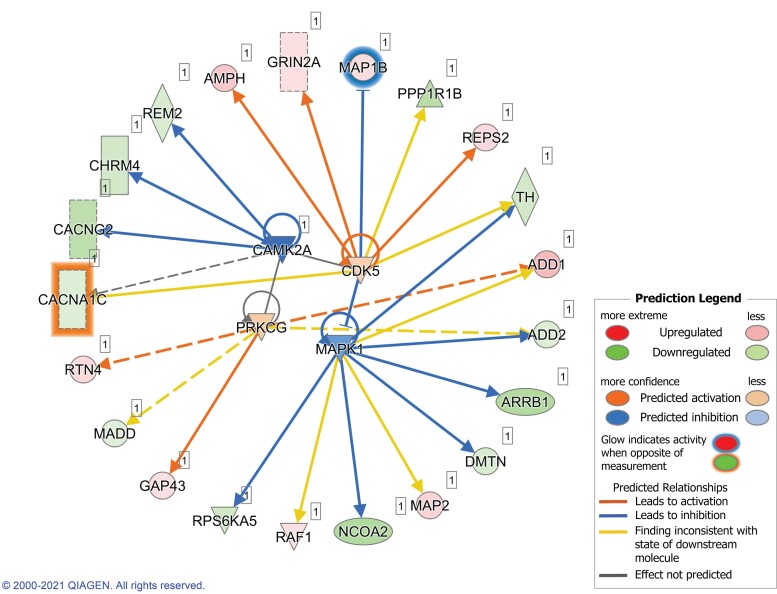
**Upstream regulators predicted by IPA.** Upstream regulators for the differentially phosphorylated proteins found in the striatum of WT versus Huntington’s disease mice under standard-housing conditions.

We also investigated which kinases and phosphatases are potentially driving the changes in phosphorylation observed in Huntington’s disease compared with WT mice. We performed the upstream analysis through IPA, selecting kinases and phosphatases as molecule types (Fig. 4). Four kinases had a calculated *Z*-score, based on the phosphorylation profiles of their targets. CAMK2A was the most significant of these, with a *P*-value of 3.0E-4 and a *Z*-score of −1.982, predicting an inhibition of the enzyme in HD_SH compared with WT_SH mice (with five of its targets being less phosphorylated). CAMK2A was itself less phosphorylated in Huntington’s disease mice at Serine 234 (Table 1). MAPK1, also known as ERK2, also appeared with a negative *Z*-score (−0.922), with six of its targets down-phosphorylated in Huntington’s disease mice. However, MAPK1 expression and phosphorylation were unaltered in HD_SH compared with WT_SH mice in our dataset. CDK5 and PRKCG (gamma subunit of PKC) were also significant Upstream Regulators, with a positive *Z*-score (0.402 and 0.447, respectively): most of their protein targets were hyperphosphorylated in HD_SH compared with WT_SH mice. Phosphorylation of PRKCG and protein expression of both CDK5 and PRKCG was found unaltered in HD_SH compared with WT_SH.

To further understand the extensive phosphorylation changes in the striatum between WT and Huntington’s disease mice in SH conditions, we also extracted phosphopeptides corresponding to kinases using the Uniprot KB keywords. We detected 278 phosphopeptides belonging to 114 kinases. Among these, 16 peptides were differentially phosphorylated (mapped to 12 kinases) between WT and Huntington’s disease mice in SH conditions (Table 1). Kinases differentially phosphorylated were mainly calcium/calmodulin regulated kinases and mitogen-activated protein kinases (Supplementary Figure 3).

Differentially phosphorylated kinases in the striatum of HD_SH compared with WT_SH. The arrows indicate the phosphorylation state in the striatum of Huntington’s disease mice compared with WT mice (↑ increased phosphorylation in Huntington’s disease; ↓ decreased phosphorylation in Huntington’s disease). Functional annotations and upstream regulator for each specific phosphorylation site were retrieved from phosphosite.org (− indicates no known functional annotation or upstream regulator).

We found that CAMKK2 phosphorylation at Serine 495 and CAMK2A phosphorylation at Threonine 337 impaired their calcium-calmodulin activation.^19,20^ Here, we observed a down-phosphorylation at these sites in Huntington’s disease compared with WT mice, which does not directly translate into an increased activity of the enzymes but might increase their probability to be activated. Specific activation phosphosites, such as the autophosphorylation site Threonine 286 on CAMK2A, were not detected in our dataset. The increased phosphorylation of PI4KB at Serine 511 observed in Huntington’s disease mice, has shown to induce a localization of the kinase in the nucleus.^21^ Also, Serine 621 phosphorylation on RAF1, found upregulated in Huntington’s disease mice, has been shown to increase the protein’s stability and prevent it from being degraded in the proteasome^22^ (Table 1).

We also detected 25 phosphopeptides mapped to 15 phosphatases. Only one of these phosphopeptides was significantly different between WT and Huntington’s disease mice in SH conditions: tyrosine-protein phosphatase non-receptor type 4 (PTPN4), at Serine 899 (no known functional annotation or upstream regulator).

### Minor changes in protein phosphorylation in the hippocampus of Huntington’s disease mice regardless of the housing conditions

In the hippocampus, we found 15 DE phosphopeptides between Huntington’s disease and WT mice in SH conditions: 12 of these were downregulated, and three were upregulated in Huntington’s disease compared with WT mice (Fig. 2C). While the number of differentially phosphorylated proteins was too low to perform a Pathway Analysis with IPA, we evaluated which protein classes these phosphoproteins belong to. We found three gene-specific transcriptional regulators: GPRASP1, TCF20 and BCL11A. Two proteins were guanine nucleotide exchange factors: PSD3 and ARHGEF17. We detected 311 phosphopeptides from 120 kinases. Only one was decreased in Huntington’s disease mice (PRKCG, Serine 373). We did not find any differentially phosphorylated phosphatases. A list of present/absent phosphopeptides in all four replicates is also available in Supplementary Table 2.

After the environmental intervention, we found fewer differences in protein phosphorylation between the two genotypes, where only four phosphopeptides were downregulated in Huntington’s disease compared with WT mice (Fig. 2D). Interestingly, in EE conditions, the microtubule-associated protein tau was found less phosphorylated at the 705 serine residue in Huntington’s disease compared with WT mice.

### No changes in protein phosphorylation in the striatum of Huntington’s disease mice housed under environmental enrichment conditions

When comparing the striatal phosphoproteome of Huntington’s disease and WT mice in the EE condition (Fig. 2B), only one phosphopeptide (MAP1A, Threonine 1633) was upregulated in Huntington’s disease compared with WT mice.

### Dysregulation in protein expression in the striatum and hippocampus of pre-motor symptomatic Huntington’s disease mice, rescued in the striatum exclusively by environmental enrichment

We also assessed whether Huntington’s disease mutation and/or EE affect protein expression in the striatum and hippocampus of 8-week-old mice.

**Figure 5:**
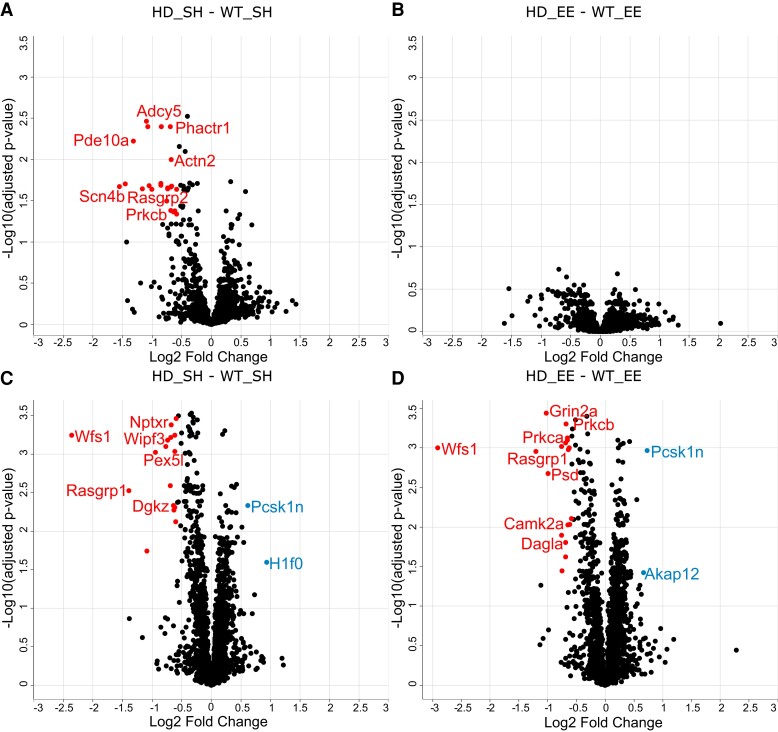
**Protein expression changes in the striatum and hippocampus of Huntington’s disease mice under standard housing versus environmental enrichment conditions.** Volcano plots of statistical significance against fold change, highlighting the significant proteins in the striatum between HD_SH and WT_SH (**A**) and between HD_EE and WT_EE (**B**); and in the hippocampus between HD_SH and WT_SH (**C**) and between HD_EE and WT_EE (**D**). Each dot represents a protein. Significant proteins (FDR 5%, FC 1.5) are highlighted in colour: left of the zero on the x-axis (red) are downregulated in HD, right of the zero on the x-avis (blue) are upregulated in HD. WT, wild-type; SH, standard housing; EE, environmental enrichment.

In the striatum, we found 24 proteins whose expression was downregulated in Huntington’s disease compared with WT mice in SH conditions (Fig. 5A). As with the phosphoproteomics dataset, we have included a list of proteins that are present/absent in all four replicates in Supplementary Table 3. Interestingly, we did not find any differences in protein expression comparing Huntington’s disease and WT mice housed under EE conditions (Fig. 5B). Among the 24 proteins found DE between Huntington’s disease and WT mice in SH, 18 had been associated with Huntington’s disease previously (Table 3).

**Table 3:** Proteins differentially expressed in the striatum of Huntington’s disease compared with WT mice under standard housing conditions

Protein symbol	Full name	−log(*P*-value)	Log2(FC)	Biological process	Evidence altered in Huntington’s disease	Mouse model (age) brain region
SCN4B	Sodium channel subunit beta-4	4.1460	−1.5547	Positive regulation sodium transport	Yes, ↓	R6/2 (4 weeks),^25^ R6/2 (12 weeks), striatum^26^
RGS9	Regulator of G-protein signalling 9	4.1450	−1.4562	G protein-coupled receptor signalling pathway	Yes, ↓	R6/2 (12 weeks), striatum^26^ R6/2 (8, 12 weeks), striatum^27^
PDE10A	cAMP and cAMP-inhibited cGMP 3′,5′,-cyclic phosphodiesterase 10A	5.9402	−1.3198	Cyclic nucleotides phosphodiesterase activity	Yes, ↓	R6/2 (8 weeks),^28^ R6/2 (12 weeks),^26^ striatum
RASGRP2	RAS guanyl-releasing protein 2	3.4442	−1.1674	Cellular response to calcium ion	Yes, ↓	R6/2 (12 weeks) striatum^26^
ADCY5	Adenylate cyclase type 5	5.2938	−1.0947	cAMP synthesis process	Yes, ↓	R6/2 (8 weeks), striatum^28^ R6/2 (8, 12 weeks), striatum^27^
ANKRD63	Ankyrin repeat domain-containing protein 63	5.4243	−1.0710	Unknown	Yes, ↓	R6/2 (12 weeks), cortex^26^
PTPN5	Tyrosine-protein phosphatase non-receptor type 5	3.8655	−1.0510	Negative regulation of MAP kinases activity	Yes (mRNA), ↓	*mRNA*: R6/1 (24 weeks), R6/2 (6 weeks), striatum^29^
ITPR1	Inositol 14,5-triphosphate receptor type1	8.1221	−1.0456	Endoplasmic reticulum calcium homeostasis	Yes, ↓	R6/2 (8, 12 weeks), striatum^27^
INF2	Inverted formin-2	3.5387	−1.0039	Actin filaments organization	Yes, ↓	R6/2 (8, 12 weeks), striatum^27^
PDE1B	Calcium/calmodulin-dependent 3′,5′-cyclic nucleotide phosphodiesterase 1B	3.8514	−0.8538	Cyclic nucleotide phosphodiesterase activity	Yes, ↓	R6/2 (8 weeks), striatum^28^
FBXL16	F-box/LRR-repeat protein 16	3.8527	−0.8501	Ubiquitin-dependent protein catabolic process	Yes, ↓	R6/2 (8, 12 weeks), striatum^27^
CACNA2D3	Voltage-dependent calcium channel subunit alpha-2/delta-3	5.4635	−0.8420	Calcium ion transport	Yes, ↓	R6/2 (8, 12 weeks), striatum^27^
TMOD1	Tropomodulin-1	3.2224	−0.7501	Actin filaments organization	No	
TRIM46	Tripartite motif-containing protein 46	3.4297	−0.7353	Cytoskeleton organization, axonogenesis	No	
CAMK4	Calcium/calmodulin-dependent protein kinase IV	3.9480	−0.7349	Positive regulation of transcription	Yes, ↓	R6/2 (6, 11 weeks), striatum^30^ R6/2 (8, 12 weeks), striatum^27^
PHACTR1	Phosphatase and actin regulator 1	5.9054	−0.6941	Actin filaments organization	Yes, ↓	R6/2 (8, 12 weeks), striatum^27^
SH2D5	SH2 domain-containing protein 5	3.0281	−0.6853	Rac-GTP levels regulation	No	
NECAB2	N-terminal EF-hand calcium-binding protein 2	3.8746	−0.6850	Intracellular calcium homeostasis	No	
ACTN2	Alpha-actinin-2	4.6819	−0.6781	F-actin cross-linking protein	Yes, ↓	R6/2 (8, 12 weeks), striatum^27^
PLD3	5′-3′ exonuclease, phospholipase D3	3.6449	−0.6711	Inflammatory response	No	
LINGO1	Leucine-rich repeat and immunoglobulin-like domain-containing nogo receptor-interacting protein 1	2.9775	−0.6365	Component of the reticulon 4 receptor	No	
MAST3	Microtubule-associated serine/threonine-protein kinase 3	2.9093	−0.5997	Cytoskeleton organization	Yes, ↓	R6/2 (8, 12 weeks), striatum^27^
PRKCB	Protein kinase C, beta type	2.9380	−0.5890	Intracellular signal transduction	Yes (mRNA), ↓	*mRNA:* R6/1 (24 weeks), R6/2 (6 weeks), striatum^29^
SRM	Spermidine synthase	3.4001	−0.5850	Spermidine synthesis	Yes (mRNA) ↓	*mRNA:* R6/1 (24 weeks), R6/2 (6 weeks) striatum^29^

List of proteins differentially expressed between HD and WT in SH in the striatum (FDR 5%, FC 1.5), sorted by log2FC. The last two columns highlight whether a downregulation in that protein’s expression has been published previously in Huntington’s disease mouse models (including at the mRNA level).

FC, fold change; negative Log2(FC) indicates the protein is downregulated in Huntington’s disease mice.

Some of the proteins we found downregulated in the striatum of Huntington’s disease mice are known to be involved in cAMP and cGMP metabolic processes, such as adenylate cyclase type 5 (ADCY5), calcium/calmodulin-dependent 3′,5′-cyclic nucleotide phosphodiesterase 1B (PDE1B) and the cyclic nucleotide phosphodiesterase 10A (PDE10). Other proteins of interest were involved in calcium homeostasis: inositol 1,4,5-triphosphate receptor type 1 (ITPR1), calcium/calmodulin-dependent protein kinase IV (CAMK4), RAS guanyl-releasing protein 2 (RASGRP2) and voltage-dependent calcium channel subunit alpha-2/delta-3 (CACNA2D3). Lastly, we found a downregulation of proteins regulating cytoskeleton organization, such as alpha-actinin-2 (ACTN2), phosphatase and actin regulator 1 (PHACTR1) and microtubule-associated serine/threonine-protein kinase 3 (MAST3). Notably, we revealed six proteins downregulated in the striatum of Huntington’s disease mice, which have not previously been described as altered in Huntington’s disease pathogenesis (Table 3). Some of these proteins also play a role in cytoskeleton organization: tropomodulin-1 (TMOD1) and tripartite motif-containing protein 46 (TRIM46). We also found a downregulation of another protein involved in intracellular calcium homeostasis: N-terminal EF-hand calcium-binding protein 2 (NECAB2).

At the protein level, we quantified 79 kinases in the striatum. Three of these were downregulated in Huntington’s disease compared with WT mice under SH conditions (MAST3, PRKCB and CAMK4) and could therefore be responsible for changes in the phosphoproteome observed between these two groups. We also measured the expression of 24 phosphatases in the striatal proteome, one of which was significantly downregulated in Huntington’s disease compared with WT mice in SH: PTPN5 (Table 3).

In the hippocampus, we found 22 proteins DE between Huntington’s disease and WT mice in SH conditions: two proteins were upregulated, and 20 proteins were downregulated in Huntington’s disease mice (Fig. 5C). We also found 22 proteins DE when comparing Huntington’s disease and WT mice under EE conditions: two proteins were upregulated in Huntington’s disease, and 20 proteins were downregulated in Huntington’s disease (Fig. 5D). Most of these differences were similar, with 15 proteins in common between the two comparisons, while seven proteins were specific to the differences between WT and Huntington’s disease mice in SH and seven were specific to the genotype differences in EE (Supplementary Figure 4). As with the striatal dataset, we have included a list of proteins that are present/absent in all four replicates in Supplementary Table 4.

Most of the proteins altered in the hippocampus between Huntington’s disease and WT mice in SH were calcium-dependent and/or involved in calcium homeostasis, including wolframin (WFS1), hippocalcin (HPCA), inositol-triphosphate kinase A (ITPKA), Ras guanyl-releasing protein 1 (RASGRP2) and protein kinase C (PRKCB). All of these were found downregulated in Huntington’s disease.

While 10 of the proteins DE in the hippocampus between Huntington’s disease and WT mice in SH had been associated with Huntington’s disease pathogenesis previously, we report 12 novel proteins, whose involvement in Huntington’s disease has not been previously reported (Table 4). These involved proteins regulating calcium and diacylglycerol signalling (SYT7, CPNE7 and DGKZ). We also reported differential expression of proteins involved in glutamate signalling: protein shisa-6 (SHISA6) and neuronal pentraxin receptor (NPTXR) were both downregulated in Huntington’s disease mice and regulate AMPA immobilization and synaptic clustering. Moreover, subunit 1 of AMPA receptor (GRIA1) was also downregulated in Huntington’s disease mice.

**Table 4:** Proteins differentially expressed in the hippocampus of Huntington’s disease compared with WT mice under standard housing conditions

Protein symbol	Full name	−log(*P*-value)	Log2(FC)	Biological process	Evidence altered in Huntington’s disease	mouse model (age), brain region
WFS1	Wolframin	5.8006	−2.3572	Calcium ion homeostasis	Yes, ↓	R6/2 (12 weeks), hippocampus^26^
RASGRP1	Ras guanyl-releasing protein 1	3.8749	−1.3971	Calcium and DAG-regulated nucleotide exchange factor	Yes, ↓	R6/2 (12 weeks), hippocampus^26^ R6/2 (8, 12 weeks), striatum^27^
RIN1	Ras and Rab interactor 1	2.7129	−1.0857	GTPase activity	Yes, ↓	R6/2 (12 weeks), hippocampus^26^
ITPKA	Inositol-triphosphate 3-kinase A	6.1320	−1.0517	Intracellular calcium signalling termination	Yes, ↓	R6/2 (12 weeks), hippocampus^26^
PSD	PH and SEC7 domain-containing protein 1	4.4205	−0.9440	Guanine nucleotide exchange factor activity	No, (Psd3 yes, ↓)	R6/2 (2, 4, 8, 8, 12 weeks), whole brain^31^
RGS14	Regulator of G-protein signalling 14	5.8757	−0.7670	G protein-coupled receptor signalling pathway	Yes, (mRNA)↓	*mRNA:* R6/2 (6, 24 weeks), striatum^29^
PRKCA	Protein kinase C	5.8220	−0.7354	Intracellular signal transduction	No (found in mHtt inclusions^32^)	
CRACDL	Capping protein-inhibiting regulator of actin-like	8.6673	−0.7245	Actin cytoskeleton maintenance	No	
CPNE7	Copine-7	4.0820	−0.6942	Cellular response to calcium ion	No,	
(Copine-5 yes: mRNA yes, ↓)	*mRNA:* R6/1 (24 weeks), striatum^33^					
WIPF3	WAS/WASL-interacting protein family member 3	5.8114	−0.6843	Actin filaments organization	Yes, ↓	R6/2 (12 weeks), hippocampus^26^
NPTXR	Neuronal pentraxin receptor	5.5566	−0.6751	Regulation of postsynaptic neurotransmitter receptor activity/assembly	No	
PRKCB	Protein kinase C, beta type	7.5843	−0.6443	Intracellular signal transduction	Yes, ↓	R6/2 (8, 12 weeks), striatum^27^
DGKZ	Diacylglycerol kinase zeta	3.5668	−0.6390	Intracellular signal transduction	No	
SYT7	Synaptotagmin-7	3.4900	−0.6338	Calcium-dependent exocytosis	No	
PEX5L	PEX5-related protein	4.4986	−0.6204	Subunit of hyperpolarization-activated cyclic nucleotide-gated channels	No	
HPCA	Neuron-specific calcium-binding protein hippocalcin	4.9671	−0.6201	Cellular response to calcium ion	Yes, (mRNA)↓	*mRNA:* R6/2 (6, 24 weeks), striatum^29,34^
MLF2	Myeloid leukaemia factor 2	3.5497	−0.6150	Unknown	Yes, (mRNA) ↓	*mRNA:* R6/2 (6, 24 weeks), striatum^29^
SHISA6	Protein shisa-6	3.3054	−0.5980	AMPA receptor clustering and activity regulation	No	
GRIA1	Glutamate ionotropic receptor AMPA type, subunit 1	6.7494	−0.5975	Ionotropic glutamate receptor activity	No	
ACTR3B	Actin-related protein 3B	5.1705	−0.5922	Actin cytoskeleton organization	No	
PCSK1N	ProSAAS/proprotein convertase 1 inhibitor	3.5691	0.6194	Neuropeptide signalling pathway	Yes, ↑	R6/2 (12 weeks), hippocampus^26^
H1-0	Histone H1	2.4924	0.9376	Chromosome condensation	No	

List of proteins differentially expressed between Huntington’s disease and WT in SH in the hippocampus (FDR 5%, FC 1.5), sorted by log2FC. The last two columns highlight if a downregulation/upregulation in that protein’s expression has been published previously in Huntington’s disease mouse models (including at the mRNA level).

FC, fold change; negative/positive Log2(FC) indicates the protein is downregulated/upregulated in Huntington’s disease mice.

We also assessed in the hippocampus whether we observed any changes in kinases and phosphatases, which might lead to differences in protein phosphorylation. In the hippocampus, we detected 102 kinases. Four of these were differently expressed in Huntington’s disease compared with WT in SH: IP3KA, KPCB, KPCA and DGKZ (Table 4). We also detected 27 phosphatases, but none were DE between the groups.

## Discussion

### Phosphoproteome dysregulation in Huntington’s disease mice in standard housing conditions

We report here, for the first time, differences in the striatal phosphoproteome of 8-week-old Huntington’s disease male mice in standard-housing conditions. Notably, these changes seem to precede the onset of motor symptoms (Supplementary Figure 5), which typically occur around 12 weeks of age in the R6/1 mouse model of Huntington’s disease used in this study.^7^ Therefore, underpinning the upstream molecular mechanisms, including the kinases and phosphatases involved, presents important therapeutic value.

Ultimately, the upstream regulation and downstream function of most of these phosphorylated sites remain unknown. Here, we investigated the functions of the proteins differentially modified, with the assumption that their activity, localization, and interactions might be affected by a change in their phosphorylation profile.

We found several differentially phosphorylated ion channels in the striatum of Huntington’s disease mice in SH conditions, including voltage-dependent ion channels, essential for the initiation and propagation of action potentials. These can be tightly regulated via phosphorylation, which can ultimately affect synaptic transmission and signal transduction.^35^ Additionally, we observed differential phosphorylation of scaffold proteins, crucial regulators of intracellular signal transduction. We also report differential phosphorylation of cytoskeleton proteins in the striatum of Huntington’s disease mice, mainly actin-binding proteins. Differential post-translational modification of actin-binding proteins, as seen in Huntington’s disease mice, can alter their interaction with actin, affecting overall actin cytoskeleton dynamics, axon growth, and neurite growth.^36^

Numerous protein-modifying enzymes were found differentially phosphorylated in the striatum of Huntington’s disease mice, including non-receptor serine/threonine protein kinases. For some of these, we were able to predict the effect of a differential phosphorylation on their activity: the decreased phosphorylation in Huntington’s disease mice of CAMK2A and CAMKK2 allows for calcium/calmodulin binding. The increased phosphorylation of PI4KB, as seen in Huntington’s disease mice, induces its localization in nuclear speckles.^21^ PI4KB functions as a signalling molecule, responsible for the phosphorylation of phosphatidylinositol into the second messenger phosphatidylinositol biphosphate, later phosphorylated into triphosphate. While the exact functions of PI4KB in the nucleus remain to be determined, phosphoinositides are known to be involved in gene transcription, mRNA processing and chromatin remodelling.^37^ RAF1, a MAPKKK acting upstream of ERK, was found to be hyperphosphorylated in Huntington’s disease mice, which translates into an increased stability of the enzyme.^22^ Concordant with our data, a previous study has found increased activation of RAF1, and its upstream regulators, in Huntington’s disease cells and mouse models.^38^

Several proteins differentially phosphorylated in Huntington’s disease were involved in the Opioid Signalling Pathway. The Opioid Signalling Pathway is not only involved in pain, but can also influence cognition and decision making,^39^ known to be impaired in clinical Huntington’s disease.^40^ More specifically, in the striatum, opioid signalling is known to interact with dopaminergic transmission, and is therefore critical for motor control in Huntington’s disease.^41^ The striatum expresses the highest levels of opioid receptors and its ligands in the brain.^42^ Although we do not expect striatal neuronal loss at this early stage of pathogenesis in these Huntington’s disease mice, a change in the phosphorylation profile of all these proteins potentially indicates a dysregulation in both the opioid and dopaminergic signalling pathways, prior to symptom onset.

Our upstream regulator analysis from IPA highlighted a potential interplay of multiple kinases responsible for the phosphoproteome shift in the striatum of Huntington’s disease mice. CAMK2A, the most significant kinase with a predicted inactivation, is a calcium/calmodulin-dependent serine/threonine protein kinase highly expressed in striatal MSNs.^43^ Once activated, it can phosphorylate and modulate targets such as NMDA and AMPA receptors,^44,45^ playing an essential role in striatal glutamatergic signalling. As such, its inhibition in the dorsal striatum leads to a loss of functional glutamatergic synapses.^46^ CAMK2A protein expression was previously found decreased in the hippocampus of fully symptomatic 10-week-old R6/2 Huntington’s disease mice.^10^ Here, while our phosphorylation data points towards an inactivation of the enzyme, we found no differences in CAMK2A protein expression between Huntington’s disease and WT mice.

Our analysis also predicted an inactivation of MAPK1 (ERK2) in the striatum of Huntington’s disease mice. The ERK pathway is involved in glutamatergic and brain-derived neurotrophic factor signalling, both of which are impaired in Huntington’s disease.^47^ Interestingly, activating ERK1/2 has shown beneficial effects in multiple cellular and animal models of Huntington’s disease.^48,49^ CDK5 and PRKCG were both predicted to be activated in the striatum of Huntington’s disease mice, concordant with previous studies, reporting aberrant activation of CDK5 in striatal cells and Huntington’s disease mouse models.^50,51^

We found few differences in the hippocampal phosphoproteome of Huntington’s disease mice (in both SH and EE conditions), compared with the extensive changes we revealed in the striatum. Our results might be explained by Huntington’s disease pathogenesis itself, characterized by a selective loss of striatal MSNs,^3^ whereby the striatum would be the first brain region to be affected. Fewer changes detected in the hippocampus might also be explained by a less homogeneous brain region, compared with the striatum. Indeed, the striatum is composed of nearly 95% of striatal projection neurons (GABAergic),^52^ while the hippocampus is more heterogeneous,^53^ and signals might cancel each other out.

In the hippocampus, we mainly observed differential phosphorylation of gene-specific transcriptional regulators, including TCF20, GPRASP1 and BCL11A. While the role of GPRASP1 on gene transcription is unknown, BCL11A haploinsufficiency has been associated with cognitive impairments.^54^

### Proteome dysregulation in Huntington’s disease mice in standard housing conditions

We found limited protein expression differences when comparing the proteome of Huntington’s disease and WT mice under SH conditions in both brain regions. The extent of these changes was expected, considering the early timepoint in Huntington’s disease pathogenesis. However, as these occur before onset of motor symptoms, they are likely critical in the development of the phenotype. As expected, we found no changes in the huntingtin protein levels at this early stage.

In the striatum, 24 proteins were found downregulated in Huntington’s disease mice compared with WT mice in SH conditions, while no proteins were found upregulated in Huntington’s disease. As previously mentioned, our study is the first one characterizing the proteome of R6/1 Huntington’s disease mice. The lack of upregulated proteins might be explained by the early timepoint in the disease progression: the downregulated proteins might be an early indication of neurodegeneration or might be a sign of sequestration into huntingtin aggregates.

Our results aligned with previously published studies, as 18 of the 24 proteins found downregulated in Huntington’s disease mice in SH had been previously associated with Huntington’s disease pathogenesis. Furthermore, 14 of these proteins are known to interact with the huntingtin protein (Supplementary Figure 6). However, here, we revealed a downregulation of these proteins prior to motor onset in Huntington’s disease mice for the first time. Indeed, most proteomic studies have been performed on R6/2 Huntington’s disease mice, which experience motor deficits from 6 to 8 weeks of age,^55^ and thus may be considered a juvenile-onset model. As 95% of Huntington’s disease patients exhibit adult onset, the R6/1 Huntington’s disease mice used in the present study provide a more accurate model of adult-onset Huntington’s disease.

As Huntington’s disease is associated with a reduced transcription of cyclic AMP early in the disease,^56^ we observed a downregulation in the expression of PDE10A and PDE1B, two cAMP/cGMP phosphodiesterases highly expressed in MSNs. ADCY5 catalyses the formation of cAMP and was also downregulated in Huntington’s disease mice. A downregulation in ADCY5 has already been highlighted in the R6/2 mouse model of Huntington’s disease, later on in the disease.^27,28^

In neurons, calcium signalling is critical for the propagation of the depolarizing signal, as well as the release of neurotransmitters at the synapse.^57^ We found a downregulation of proteins involved in calcium homeostasis in the striatum, including a voltage-gated calcium channel subunit (CACNA2D3), a neuronal calcium sensor (NECAB2), a regulator of calcium release from endoplasmic reticulum (ITPR1) and a calcium and diacylglycerol dependent protein kinase (PRKCB).

In the present study, we also revealed a downregulation in the striatum of Huntington’s disease mice of six novel proteins (Table 3). The phospholipase D3 (PLD3), found in neuronal lysosomes, was found reduced in the striatum of Huntington’s disease mice. Interestingly, a reduced function of the protein has been correlated with a faster rate of cognitive decline in Alzheimer’s disease patients.^58^ Further studies would be needed to underpin its involvement in Huntington’s disease-associated cognitive deficits. Tropomodulin 1 (TMOD1), an actin-binding protein essential for synapse formation,^59^ was found downregulated in the striatum of Huntington’s disease mice. Altered expression of TMOD1 has been found in several brain disorders, such as epilepsy^60^ and Down syndrome^61^ but not been described in Huntington’s disease before. Decreased levels of SH2 domain-containing protein 5, as seen in the striatum of Huntington’s disease mice, has been associated with impaired neurogenesis and synaptogenesis.^62^

Together, the proteins found downregulated in the striatum of Huntington’s disease mice under SH conditions prior to motor symptoms indicate dysregulation in calcium signalling, destabilization of the cytoskeleton and neuronal cell loss. Our results are concordant with previous studies, and will guide further investigations exploring the involvement of associated molecular and cellular processes in Huntington’s disease pathogenesis.

In the hippocampus, we reported 22 proteins DE between WT and Huntington’s disease mice when housed in SH conditions. Several proteins we found dysregulated in the hippocampus were involved in calcium signalling, including wolframin (WFS1, which maintains ER calcium homeostasis), and hippocalcin (HPCA), a neuronal calcium sensor. We also report a downregulation of proteins involved in glutamatergic signalling in the hippocampus of Huntington’s disease mice: SHISA6, responsible for AMPA-type glutamate receptor immobilization at postsynaptic density, and glutamate receptor 1 (GRIA1, AMPA type). AMPA receptors are known to mediate most of the fast excitatory synaptic transmission, playing an important role in long-term potentiation and long-term depression of synaptic transmission.^63^

### The effect of the environment on the proteome and phosphoproteome

Our analysis also investigated a potential effect of EE, from 4 to 8 weeks of age, on the phosphoproteome and proteome of Huntington’s disease mice and their WT littermates. EE has previously been shown to improve cognitive and motor performance in Huntington’s disease mice, as well as to alleviate Huntington’s disease-related motor, affective and cognitive symptoms.^64^ Our experiment was designed with an environmental intervention initiated in young mice, as the brain is more sensitive to an environmental modulation early in development,^64^ and the most striking therapeutic effects of EE on Huntington’s disease mice have involved delay in disease onset.^13,64^ The behavioural outcomes of such an early and short environmental intervention had not yet been investigated. Here, contrary to previously published studies,^64^ we found no effects of EE on the motor performance of 8-week-old mice (Supplementary Figure 5). These results on Huntington’s disease mice could be explained by the early timepoint in the disease progression, where an improvement in motor performance cannot be seen before onset of Huntington’s disease-associated motor symptoms.

Although not seen at the behavioural level (as the tissues were collected at 8 weeks of age which is prior to motor onset in these Huntington’s disease mice), it is possible that such early environmental intervention leads to long-lasting effects, including those acting on a molecular level. Indeed, when mice were housed in EE conditions, we found no significant differences in protein phosphorylation in the striatum between Huntington’s disease mice and their WT littermates (apart from one phosphopeptide). The lack of protein phosphorylation changes in the striatum of Huntington’s disease and WT mice after an EE intervention could be explained by the rescue of certain protein kinases and phosphatases, at the protein level. Indeed, while PKC, MAST3, CAMK4 and PTPN5 were found downregulated in SH conditions, their expression was not significantly changed between the two genotypes in EE conditions. Phosphorylation changes were also reduced in the hippocampus when both genotypes were housed in EE.

Interestingly, in the hippocampus, we revealed a reduced MAPT (tau protein) phosphorylation at amino-acid residue 705 when comparing Huntington’s disease versus WT mice housed in EE (Fig. 2D). This finding is in contrast to our recent study which found an increased tau phosphorylation in the cortex of 8-week-old R6/1 Huntington’s disease mice when housed in SH conditions.^8^ However, this could reflect a potential beneficial effect of EE in Huntington’s disease mice, whereby EE could have a different effect on WT versus Huntington’s disease mice.

At the protein level, we found no differences between the striatal proteome of Huntington’s disease and WT mice in EE conditions. In the hippocampus, the same number of DE proteins was found when comparing Huntington’s disease and WT in EE conditions and in SH conditions. However, the proteins DE were partly different in the two conditions, suggesting that EE might have a different effect on the hippocampus of Huntington’s disease and WT mice.

Previous EE interventions have been shown to reduce the size of polyglutamine-expanded huntingtin aggregates in Huntington’s disease mice.^65^ We can hypothesize that the reduced aggregate size correlates with a delay of protein sequestration into the inclusion bodies, therefore found with an unchanged concentration in the soluble fraction in enrichment conditions. Indeed, most of the proteins found downregulated in the striatum of Huntington’s disease mice in SH conditions, prior to motor symptoms, are known to interact with huntingtin protein (Supplementary Figure 6).

One limitation of the present study is that it is unknown which component of EE is driving these changes in protein phosphorylation and protein expression (i.e. physical activity, sensory simulation, cognitive activity, etc.). Furthermore, it could involve multiple components acting in synergy. This is beyond the scope of the current study, however further experiments investigating specific components of EE would help address the underlying mechanisms. Additionally, the environmental paradigm used in this study presents limitations regarding its translation to the clinic. The SH condition, in which we observed extensive dysregulations in protein phosphorylation, would refer to a deprivation state in humans, whereas EE is likely to be closely to a ‘normal’ (or at least average) level of sensorimotor stimulation and physical activity in humans.

When comparing WT in SH versus WT in EE, and Huntington’s disease in SH versus Huntington’s disease in EE, we did not find any significant proteins or phosphopeptides in both brain regions (Supplementary Figures 7–10). Previous studies have reported specific protein expression and protein phosphorylation changes in WT rodents and Huntington’s disease mouse models after a EE intervention.^66–69^ However, the enrichment protocol used in these studies, as well as the duration of the intervention and the age of the animals at the start of the intervention vary with our present study. These factors are known to affect the outcome of such interventions.^70^ The stringent statistical thresholds (i.e. FDR 5% and FC 1.5) could also help explain the results, as our study might have been underpowered to detect a smaller effect of environmental intervention, compared with the Huntington’s disease transgene effect. The environmental intervention could also have a different effect on WT and Huntington’s disease mice, leading to significant changes between the two genotypes only in standard-housing conditions. The correlation among samples was similar in each group (see Pearson’s correlation in methods), thus a potential higher variability in EE samples does not explain the lack of significant results.

In the current study, we found that a protein phosphorylation dysregulation occurs in the striatum of Huntington’s disease male mice at a very early stage of pathogenesis, prior to onset of motor deficits. This was associated with minimal changes in protein expression. Following up on our previous findings revealing a phosphoproteome dysregulation occurring in the cortex of pre-symptomatic Huntington’s disease male mice,^8^ the present study only investigated protein and phosphorylation changes in male mice. Additional proteomics and phosphoproteomics experiments using Huntington’s disease female brain samples remain to be conducted. Comparing male versus female data in a future study would be informative.

## Conclusion

Here, we have identified new leads to develop novel therapeutics for Huntington’s disease, by targeting the phosphoproteome. We also report the outcome of a 4-week EE intervention, which was able to rescue these changes observed in the striatum of Huntington’s disease mice, both at the protein expression and protein phosphorylation levels. While the exact molecular mechanisms of EE are unclear, this intervention has previously shown beneficial effects on Huntington’s disease mice, on cognitive, affective and motor deficits, as well as neurodegeneration. Our current study reveals the effects of EE on protein phosphorylation in Huntington’s disease mice, which will inform the future development of novel therapeutic approaches, including enviromimetics.

## Supplementary Material

fcac305_Supplementary_Data

## Data Availability

The mass spectrometry proteomics data have been deposited to the ProteomeXchange Consortium via the PRIDE^18^ partner repository with the dataset identifier PXD032205.

## Abbreviations

CAG =: cytosine-adenine-guanine
EE =: environmental enrichment
FC =: fold change
FDR =: false discovery rate
HD =: Huntington’s disease
IPA =: ingenuity pathway analysis
LC-MS/MS =: liquid chromatography with tandem mass spectrometry
MSNs =: medium spiny neurons
SH =: standard housing
WT =: wild-type

## References

[1] MacDonald ME , Ambrose CM, Duyao MP, et al A novel gene containing a trinucleotide repeat that is expanded and unstable on Huntington’s disease chromosomes. Cell. 1993;72(6):971–983. 8458085 10.1016/0092-8674(93)90585-e

[2] Perutz MF , Johnson T, Suzuki M, Finch JT. Glutamine repeats as polar zippers: Their possible role in inherited neurodegenerative diseases. Proc Natl Acad Sci USA. 1994;91(12):5355–5358. 8202492 10.1073/pnas.91.12.5355 PMC43993

[3] Waldvogel HJ , Kim EH, Tippett LJ, Vonsattel JPG, Faull RLM. The neuropathology of Huntington’s disease. Curr Top Behav Neurosci. 2015;22:33–80. 25300927 10.1007/7854_2014_354

[4] Cardoso F . Huntington disease and other choreas. Neurol Clin. 2009;27(3):719–736. 19555828 10.1016/j.ncl.2009.04.001

[5] Renoir T , Zajac MS, Du X, et al Sexually dimorphic serotonergic dysfunction in a mouse model of Huntington’s disease and depression. PLoS ONE. 2011;6(7):e22133. 21760962 10.1371/journal.pone.0022133 PMC3132782

[6] Mo C , Renoir T, Pang TYC, Hannan AJ. Short-term memory acquisition in female Huntington’s disease mice is vulnerable to acute stress. Behav Brain Res. 2013;253:318–322. 23916759 10.1016/j.bbr.2013.07.041

[7] Pang TYC , Stam NC, Nithianantharajah J, Howard ML, Hannan AJ. Differential effects of voluntary physical exercise on behavioral and brain-derived neurotrophic factor expression deficits in Huntington’s disease transgenic mice. Neuroscience. 2006;141(2):569–584. 16716524 10.1016/j.neuroscience.2006.04.013

[8] Mees I , Tran H, Roberts A, et al Quantitative phosphoproteomics reveals extensive protein phosphorylation dysregulation in the cerebral cortex of Huntington’s disease mice prior to onset of symptoms. Mol Neurobiol. 2022;59(4):2456–2471. 35083661 10.1007/s12035-021-02698-y

[9] Blum D , Herrera F, Francelle L, et al Mutant huntingtin alters tau phosphorylation and subcellular distribution. Hum Mol Genet. 2015;24(1):76–85. 25143394 10.1093/hmg/ddu421

[10] Gratuze M , Noël A, Julien C, et al Tau hyperphosphorylation and deregulation of calcineurin in mouse models of Huntington’s disease. Hum Mol Genet. 2015;24(1):86–99. 25205109 10.1093/hmg/ddu456

[11] Fernández-Nogales M , Hernández F, Miguez A, et al Decreased glycogen synthase kinase-3 levels and activity contribute to Huntington’s disease. Hum Mol Genet. 2015;24(17):5040–5052. 26082469 10.1093/hmg/ddv224

[12] Schmidt ME , Caron NS, Aly AE, et al DAPK1 promotes extrasynaptic GluN2B phosphorylation and striatal spine instability in the YAC128 mouse model of Huntington disease. Front Cell Neurosci. 2020;14:590569. 33250715 10.3389/fncel.2020.590569 PMC7674490

[13] Van Dellen A , Blakemore C, Deacon R, York D, Hannan AJ. Delaying the onset of Huntington’s in mice. Nature. 2000;404(6779):721–722. 10783874 10.1038/35008142

[14] Nithianantharajah J , Barkus C, Murphy M, Hannan AJ. Gene-environment interactions modulating cognitive function and molecular correlates of synaptic plasticity in Huntington’s disease transgenic mice. Neurobiol Dis. 2008;29(3):490–504. 18165017 10.1016/j.nbd.2007.11.006

[15] Pang TYC , Du X, Zajac MS, Howard ML, Hannan AJ. Altered serotonin receptor expression is associated with depression-related behavior in the R6/1 transgenic mouse model of Huntington’s disease. Hum Mol Genet. 2009;18(4):753–766. 19008301 10.1093/hmg/ddn385

[16] Dubois C , Kong G, Tran H, et al Small non-coding RNAs are dysregulated in Huntington’s disease transgenic mice independently of the therapeutic effects of an environmental intervention. Mol Neurobiol. 2021;58(7):3308–3318. 33675499 10.1007/s12035-021-02342-9

[17] Cox J , Mann M. MaxQuant enables high peptide identification rates, individualized p.p.b.-range mass accuracies and proteome-wide protein quantification. Nat Biotechnol. 2008;26(12):1367–1372. 19029910 10.1038/nbt.1511

[18] Perez-Riverol Y , Bai J, Bandla C, et al The PRIDE database resources in 2022: A hub for mass spectrometry-based proteomics evidences. Nucleic Acids Res. 2022;50(D1):D543–D552. 34723319 10.1093/nar/gkab1038 PMC8728295

[19] Langendorf CG , O’Brien MT, Ngoei KRW, et al CaMKK2 is inactivated by cAMP-PKA signaling and 14-3-3 adaptor proteins. J Biol Chem. 2020;295(48):16239–16250. 32913128 10.1074/jbc.RA120.013756 PMC7705300

[20] Wang P , Mei F, Hu J, et al PTENα modulates CaMKII signaling and controls contextual fear memory and spatial learning. Cell Rep. 2017;19(12):2627–2641. 28636948 10.1016/j.celrep.2017.05.088

[21] Szivak I , Lamb N, Heilmeyer LMG. Subcellular localization and structural function of endogenous phosphorylated phosphatidylinositol 4-kinase (PI4K92). J Biol Chem. 2006;281(24):16740–9. 16606619 10.1074/jbc.M511645200

[22] Noble C , Mercer K, Hussain J, et al CRAF autophosphorylation of serine 621 is required to prevent its proteasome-mediated degradation. Mol Cell. 2008;31(6):862–872. 18922468 10.1016/j.molcel.2008.08.026 PMC2640467

[23] Nguyen N , Olivas TJ, Mires A, et al The insufficiency of ATG4A in macroautophagy. J Biol Chem. 2020;295(39):13584–13600. 32732290 10.1074/jbc.RA120.013897 PMC7521654

[24] Li F , Kozono D, Deraska P, et al CHK1 inhibitor blocks phosphorylation of FAM122A and promotes replication stress. Mol Cell. 2020;80(3):410–422.e6. 33108758 10.1016/j.molcel.2020.10.008 PMC7761918

[25] Oyama F , Miyazaki H, Sakamoto N, et al Sodium channel β4 subunit: Down-regulation and possible involvement in neuritic degeneration in Huntington’s disease transgenic mice. J Neurochem. 2006;98(2):518–529. 16805843 10.1111/j.1471-4159.2006.03893.x

[26] Skotte NH , Andersen JV, Santos A, et al Integrative characterization of the R6/2 mouse model of Huntington’s disease reveals dysfunctional astrocyte metabolism. Cell Rep. 2018;23(7):2211–2224. 29768217 10.1016/j.celrep.2018.04.052

[27] Diaz-Castro B , Gangwani MR, Yu X, Coppola G, Khakh BS. Astrocyte molecular signatures in Huntington’s disease. Sci Transl Med. 2019;11(514):eaaw8546. 31619545 10.1126/scitranslmed.aaw8546

[28] Hosp F , Gutiérrez-Ángel S, Schaefer MH, et al Spatiotemporal proteomic profiling of Huntington’s disease inclusions reveals widespread loss of protein function. Cell Rep. 2017;21(8):2291–2303. 29166617 10.1016/j.celrep.2017.10.097 PMC5714591

[29] Kuhn A , Goldstein DR, Hodges A, et al Mutant huntingtin’s effects on striatal gene expression in mice recapitulate changes observed in human Huntington’s disease brain and do not differ with mutant huntingtin length or wild-type huntingtin dosage. Hum Mol Genet. 2007;16(15):1845–1861. 17519223 10.1093/hmg/ddm133

[30] Deckel AW , Elder R, Fuhrer G. Biphasic developmental changes in Ca^2+^/calmodulin-dependent proteins in R6/2 Huntington’s disease mice. Neuroreport. 2002;13(5):707–711. 11973475 10.1097/00001756-200204160-00034

[31] Zabel C , Mao L, Woodman B, et al A large number of protein expression changes occur early in life and precede phenotype onset in a mouse model for Huntington disease. Mol Cell Proteomics. 2009;8(4):720–734. 19043139 10.1074/mcp.M800277-MCP200 PMC2667354

[32] Zemskov EA , Jana NR, Kurosawa M, et al Pro-apoptotic protein kinase Cδ is associated with intranuclear inclusions in a transgenic model of Huntington’s disease. J Neurochem. 2003;87(2):395–406. 14511117 10.1046/j.1471-4159.2003.02002.x

[33] Desplats PA , Kass KE, Gilmartin T, et al Selective deficits in the expression of striatal-enriched mRNAs in Huntington’s disease. J Neurochem. 2006;96:743–757. 16405510 10.1111/j.1471-4159.2005.03588.x

[34] Luthi-Carter R , Strand A, Peters NL, et al Decreased expression of striatal signaling genes in a mouse model of Huntington’s disease. Hum Mol Genet. 2000;9:1259–1271. 10814708 10.1093/hmg/9.9.1259

[35] Ismailov II , Benos DJ. Effects of phosphorylation on ion channel function. Kidney Int. 1995;48(4):1167–1179. 8569078 10.1038/ki.1995.400

[36] Varland S , Vandekerckhove J, Drazic A. Actin post-translational modifications: The Cinderella of cytoskeletal control. Trends Biochem Sci. 2019;44(6):502–516. 30611609 10.1016/j.tibs.2018.11.010

[37] Jacobsen RG , Mazloumi Gavgani F, Edson AJ, Goris M, Altankhuyag A, Lewis AE. Polyphosphoinositides in the nucleus: Roadmap of their effectors and mechanisms of interaction. Adv Biol Regul. 2019;72:7–21. 31003946 10.1016/j.jbior.2019.04.001

[38] Miller JP , Yates BE, Al-Ramahi I, et al A genome-scale RNA-interference screen identifies RRAS signaling as a pathologic feature of Huntington’s disease. PLoS Genet. 2012;8(11):e1003042. 23209424 10.1371/journal.pgen.1003042 PMC3510027

[39] van Steenbergen H , Eikemo M, Leknes S. The role of the opioid system in decision making and cognitive control: A review. Cogn Affect Behav Neurosci. 2019;19(3):435–458. 30963411 10.3758/s13415-019-00710-6 PMC6599188

[40] Stout JC , Rodawalt WC, Siemers ER. Risky decision making in Huntington’s disease. J Int Neuropsychol Soc. 2001;7(1):92–101. 11253845 10.1017/s1355617701711095

[41] Sgroi S , Tonini R. Opioidergic modulation of striatal circuits, implications in Parkinson’s disease and levodopa induced dyskinesia. Front Neurol. 2018;9:524. 30026724 10.3389/fneur.2018.00524 PMC6041411

[42] Mansour A , Hoversten MT, Taylor LP, Watson SJ, Akil H. The cloned μ, δ and κ receptors and their endogenous ligands: Evidence for two opioid peptide recognition cores. Brain Res. 1995;700:89–98. 8624732 10.1016/0006-8993(95)00928-j

[43] Erondu NE , Kennedy MB. Regional distribution of type II Ca^2+^/calmodulin-dependent protein kinase in rat brain. J Neurosci. 1985;5(12):3270–3277. 4078628 10.1523/JNEUROSCI.05-12-03270.1985 PMC6565219

[44] Kristensen AS , Jenkins MA, Banke TG, et al Mechanism of CaMKII regulation of AMPA receptor gating. Nat Neurosci. 2011;14(6):727–735. 21516102 10.1038/nn.2804 PMC3102786

[45] Strack S , McNeill RB, Colbran RJ. Mechanism and regulation of calcium/calmodulin-dependent protein kinase II targeting to the NR2B subunit of the N-methyl-D-aspartate receptor. J Biol Chem. 2000;275:23798–23806. 10764765 10.1074/jbc.M001471200

[46] Klug JR , Mathur BN, Kash TL, et al Genetic inhibition of CaMKII in dorsal striatal medium spiny neurons reduces functional excitatory synapses and enhances intrinsic excitability. PLoS ONE. 2012;7:e45323. 23028932 10.1371/journal.pone.0045323 PMC3448631

[47] Bodai L , Marsh JL. A novel target for Huntington’s disease: ERK at the crossroads of signaling. BioEssays. 2012;34:142–1148. 22334892 10.1002/bies.201100116 PMC3711381

[48] Maher P , Dargusch R, Bodai L, Gerard PE, Purcell JM, Lawrence Marsh J. Erk activation by the polyphenols fisetin and resveratrol provides neuroprotection in multiple models of Huntington’s disease. Hum Mol Genet. 2011;20:261–2270. 20952447 10.1093/hmg/ddq460 PMC3005900

[49] Apostol BL , Illes K, Pallos J, et al Mutant huntingtin alters MAPK signaling pathways in PC12 and striatal cells: ERK1/2 protects against mutant huntingtin-associated toxicity. Hum Mol Genet. 2006;15:273–2285. 16330479 10.1093/hmg/ddi443

[50] Paoletti P , Vila I, Rifé M, Lizcano JM, Alberch J, Ginés S. Dopaminergic and glutamatergic signaling crosstalk in Huntington’s disease neurodegeneration: The role of p25/cyclin-dependent kinase 5. J Neurosci. 2008;28:10090–110101. 18829967 10.1523/JNEUROSCI.3237-08.2008 PMC6671267

[51] Alvarez-Periel E , Puigdellívol M, Brito V, et al Cdk5 contributes to Huntington’s disease learning and memory deficits via modulation of brain region-specific substrates. Mol Neurobiol. 2018;55:6250–66268. 29288339 10.1007/s12035-017-0828-4

[52] Gerfen CR , Scott Young W. Distribution of striatonigral and striatopallidal peptidergic neurons in both patch and matrix compartments: An in situ hybridization histochemistry and fluorescent retrograde tracing study. Brain Res. 1988;460:161–1167. 2464402 10.1016/0006-8993(88)91217-6

[53] Wheeler DW , White CM, Rees CL, Komendantov AO, Hamilton DJ, Ascoli GA. Hippocampome.org: A knowledge base of neuron types in the rodent hippocampus. Elife. 2015;4:e09960 10.7554/eLife.09960 PMC4629441 26402459

[54] Dias C , Estruch SB, Graham SA, et al BCL11A Haploinsufficiency causes an intellectual disability syndrome and dysregulates transcription. Am J Hum Genet. 2016;99:253–2274. 27453576 10.1016/j.ajhg.2016.05.030 PMC4974071

[55] Carter RJ , Lione LA, Humby T, et al Characterization of progressive motor deficits in mice transgenic for the human Huntington’s disease mutation. J Neurosci. 1999;19:3248–33257. 10191337 10.1523/JNEUROSCI.19-08-03248.1999 PMC6782264

[56] Gines S , Seong IS, Fossale E, et al Specific progressive cAMP reduction implicates energy deficit in presymptomatic Huntington’s disease knock-in mice. Hum Mol Genet. 2003;12(5):497–508. 12588797 10.1093/hmg/ddg046

[57] Brini M , Calì T, Ottolini D, Carafoli E. Neuronal calcium signaling: Function and dysfunction. Cell Mol Life Sci. 2014;71:2787–22814. 24442513 10.1007/s00018-013-1550-7 PMC11113927

[58] Nackenoff AG , Hohman TJ, Neuner SM, et al PLD3 Is a neuronal lysosomal phospholipase D associated with β-amyloid plaques and cognitive function in Alzheimer’s disease. PLoS Genet. 2021;17:e1009406. 33830999 10.1371/journal.pgen.1009406 PMC8031396

[59] Omotade OF , Rui Y, Lei W, et al Tropomodulin isoform-specific regulation of dendrite development and synapse formation. J Neurosci. 2018;38:10271–110285. 30301754 10.1523/JNEUROSCI.3325-17.2018 PMC6262146

[60] Sussman MA , Sakhi S, Tocco G, et al Neural tropomodulin: Developmental expression and effect of seizure activity. Dev Brain Res. 1994;80:45–453. 7955359 10.1016/0165-3806(94)90088-4

[61] Sun Y , Dierssen M, Toran N, Pollak DD, Chen WQ, Lubec G. A gel-based proteomic method reveals several protein pathway abnormalities in fetal down syndrome brain. J Proteomics. 2011;74:547–5557. 21262400 10.1016/j.jprot.2011.01.009

[62] Gray EJ , Petsalaki E, James DA, et al Src homology 2 domain containing protein 5 (SH2D5) binds the breakpoint cluster region protein, BCR, and regulates levels of Rac1-GTP. J Biol Chem. 2014;289(51):35397–35408. 25331951 10.1074/jbc.M114.615112 PMC4271225

[63] Zhang H , Bramham CR. Bidirectional dysregulation of AMPA receptor-mediated synaptic transmission and plasticity in brain disorders. Front Synaptic Neurosci. 2020;12:26. 32754026 10.3389/fnsyn.2020.00026 PMC7366028

[64] Nithianantharajah J , Hannan AJ. Enriched environments, experience-dependent plasticity and disorders of the nervous system. Nat Rev Neurosci. 2006;7(9):697–709. 16924259 10.1038/nrn1970

[65] Benn CL , Luthi-Carter R, Kuhn A, et al Environmental enrichment reduces neuronal intranuclear inclusion load but has no effect on messenger RNA expression in a mouse model of Huntington disease. J Neuropathol Exp Neurol. 2010;69(8):817–827. 20613636 10.1097/NEN.0b013e3181ea167f

[66] Spires TL , Grote HE, Varshney NK, et al Environmental enrichment rescues protein deficits in a mouse model of Huntington’s disease, indicating a possible disease mechanism. J Neurosci. 2004;24(9):2270–2276. 14999077 10.1523/JNEUROSCI.1658-03.2004 PMC6730435

[67] Glass M , Van Dellen A, Blakemore C, Hannan AJ, Faull RLM. Delayed onset of Huntington’s disease in mice in an enriched environment correlates with delayed loss of cannabinoid CB1 receptors. Neuroscience. 2004;123(1):207–212. 14667455 10.1016/s0306-4522(03)00595-5

[68] Fan X , Li D, Zhang Y, Green TA. Differential phosphoproteome regulation of nucleus accumbens in environmentally enriched and isolated rats in response to acute stress. PLoS ONE. 2013;8(11):e79893 10.1371/journal.pone.0079893 PMC3838351 24278208

[69] Hu YS , Long N, Pigino G, Brady ST, Lazarov O. Molecular mechanisms of environmental enrichment: Impairments in Akt/GSK3β, neurotrophin-3 and CREB signaling. PLoS ONE. 2013;8(5):e64460. 23700479 10.1371/journal.pone.0064460 PMC3660250

[70] Amaral OB , Vargas RS, Hansel G, Izquierdo I, Souza DO. Duration of environmental enrichment influences the magnitude and persistence of its behavioral effects on mice. Physiol Behav. 2008;93(1–2):388–394. 17949760 10.1016/j.physbeh.2007.09.009

