# Supplementary material for: Phosphoproteomic dysregulation in Huntington’s disease mice is rescued by environmental enrichment
Source: Brain Commun. 2022 Nov 21;4(6):fcac305. doi: 10.1093/braincomms/fcac305 (PMC9746689; doi:10.1093/braincomms/fcac305)
Supplement: fcac305_Supplementary_Data [file fcac305_supplementary_data.pdf]

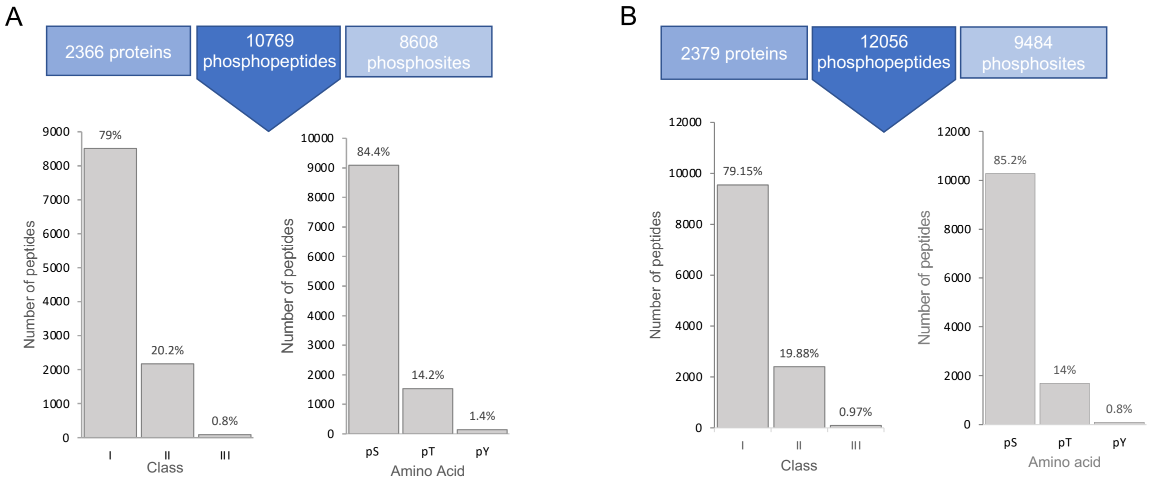

Supplementary Figure 1: Phosphoproteome characterization

Number of phosphoproteins, phosphopeptides and phosphosites identified across all samples in the striatum (A) and hippocampus (B). Left: percentage of phosphopeptides identified in class I (Phosphate localization confidence > 75%), class II (confidence between 50% and 75%) and class III (confidence < 50%). Right: percentage of phosphopeptides with a phosphorylated Serine (pS), Threonine (pT) and Tyrosine (pY) residue.

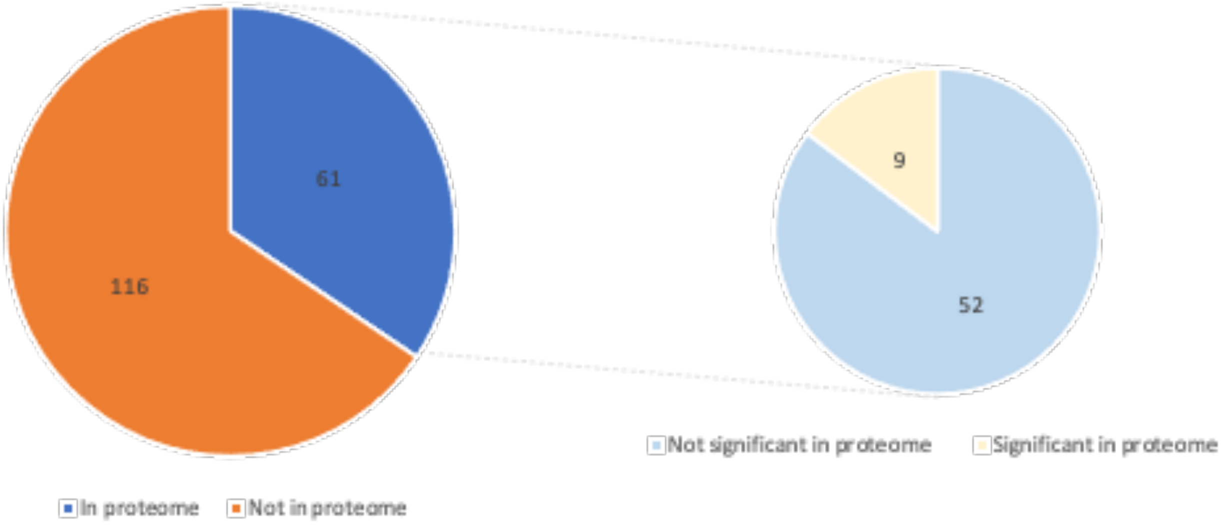

Protein ID	Log2FC Protein	Log2FC Phosphorylation site
ANKR63	-1.07099	-1.358597501
NECAB2	-0.685026	-1.394941139
ADCY5	-1.09467	-1.109019597
ITPR1	-1.04558	-2.253852503
PRKCB	-0.589038	-0.634583092
RGS9	-1.45623	-1.603411357
PDE10A	-1.31982	-1.863530858
RASGRP2	-1.16743	-1.659315332,<br>-1.940964723,<br>-2.222796623
MAST3	-0.599677	-0.96770153

Supplementary Figure 2: Proteins and phosphorylation sites downregulated in HD striatum

Number of significant phosphopeptides that were also measured at the protein level. List of proteins significantly downregulated at the protein level, and whose phosphorylation downregulated in the striatum of HD_SH mice compared to WT_SH.

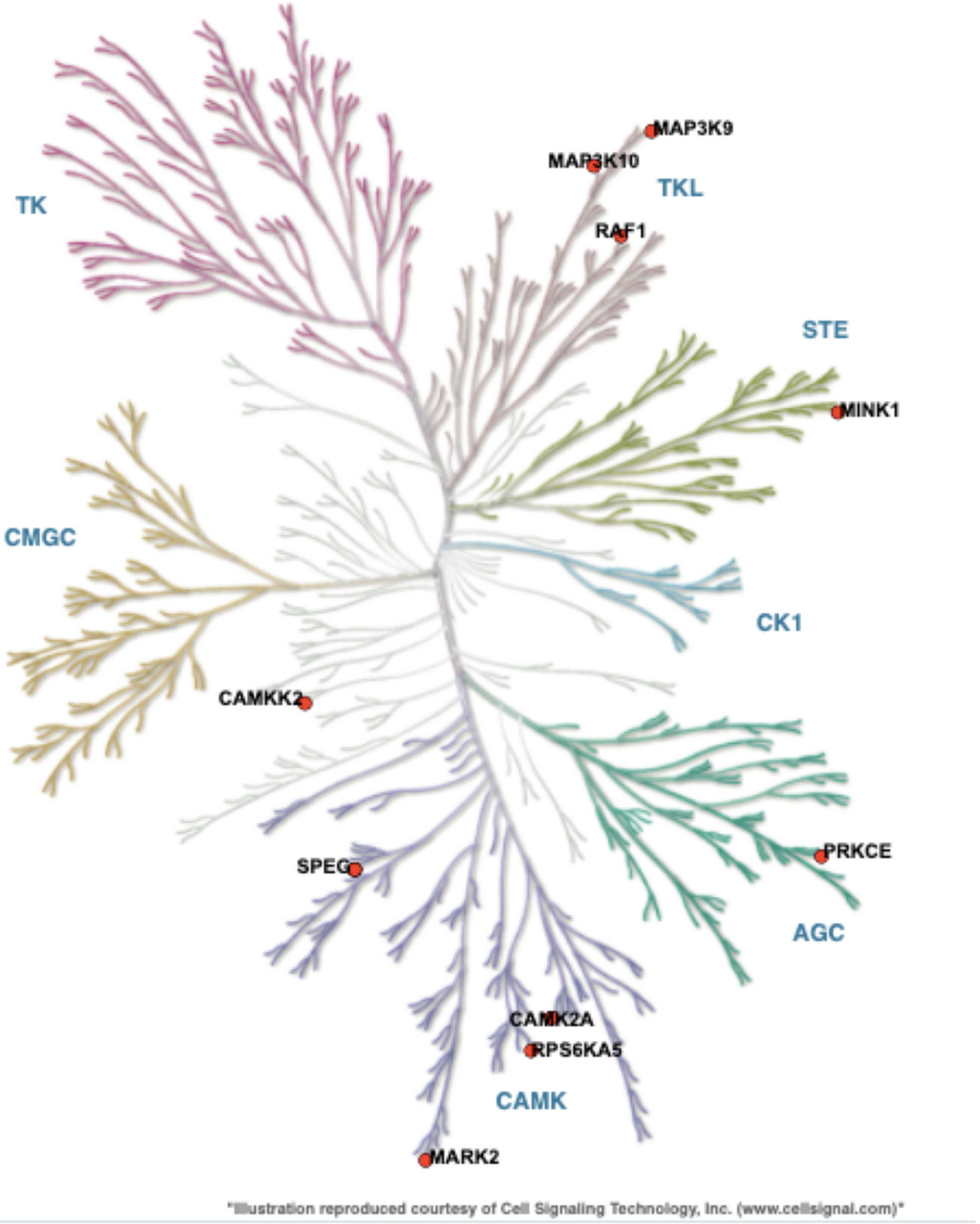

Supplementary Figure 3: Kinases differentially phosphorylated in HD striatum

Using KinMap we located the differentially phosphorylated kinases between WT-SH and HD-SH in the striatum on the Human Kinome Tree (highlighted in red). TK, Tyrosine Kinases; TKL, Tyrosine kinase-like; STE, MAPK cascade families; CK1, CK1, TTBK (Tau-tubulin kinase) and VRK (vaccinia-related kinase) families; AGC, protein kinase A,G,C families; CAMK, calmodulin/calcium regulated kinases and some non-calcium regulated families; CMGC, CDK, GSK3, MAPK, CLK kinase families (Kinhub.org)

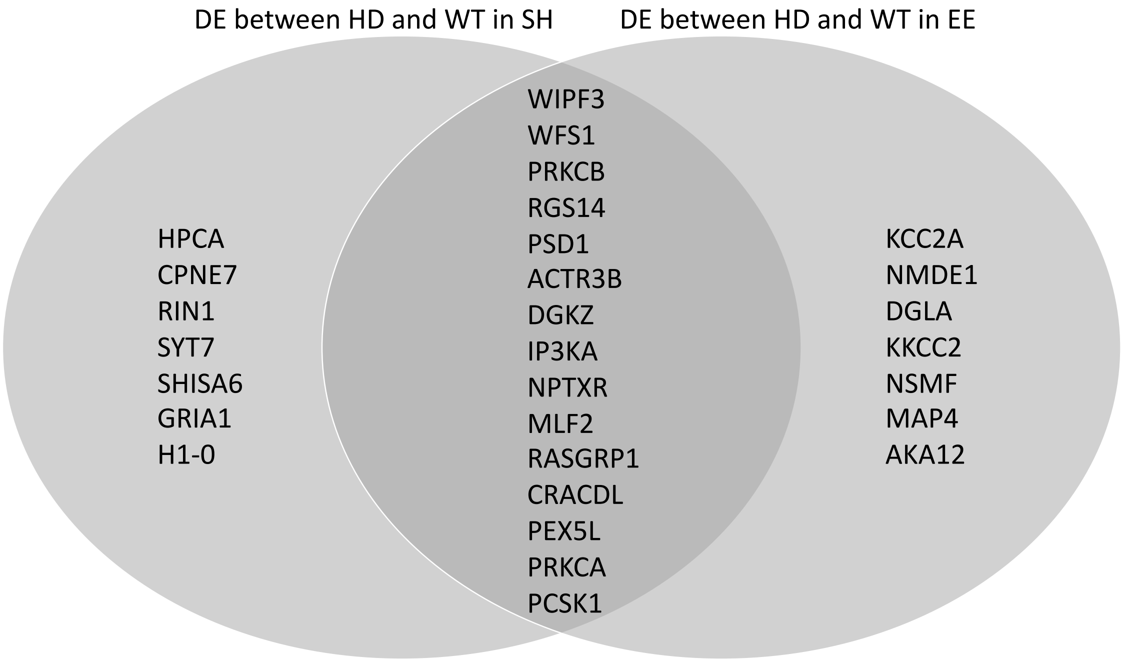

Supplementary Figure 4: Differentially expressed proteins in the HD hippocampus

Venn Diagram of DE proteins in the hippocampus between HD and WT in SH, and between WT and HD in EE (right). The proteins in the middle are DE between the genotypes in both conditions.

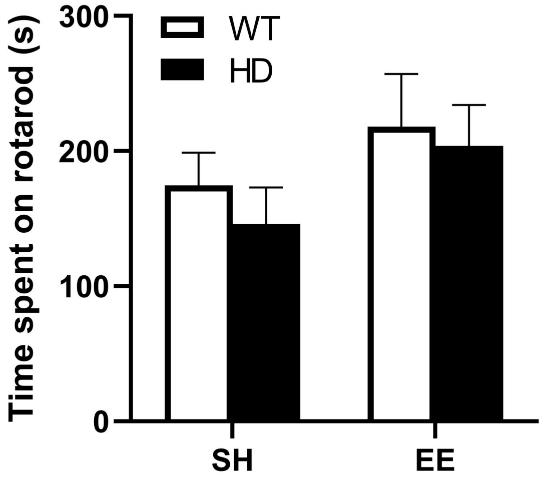

Supplementary Figure 5: Rotarod task

A two-way ANOVA analysis revealed no significant effect of the HD genotype (p=0.4918), environmental enrichment (p=0.1138) nor interaction between genotype and housing (p=0.8192) on the 8-week-old male mice performance at the rotarod task. Error bars represent mean +- SEM. N=6 mice per group.

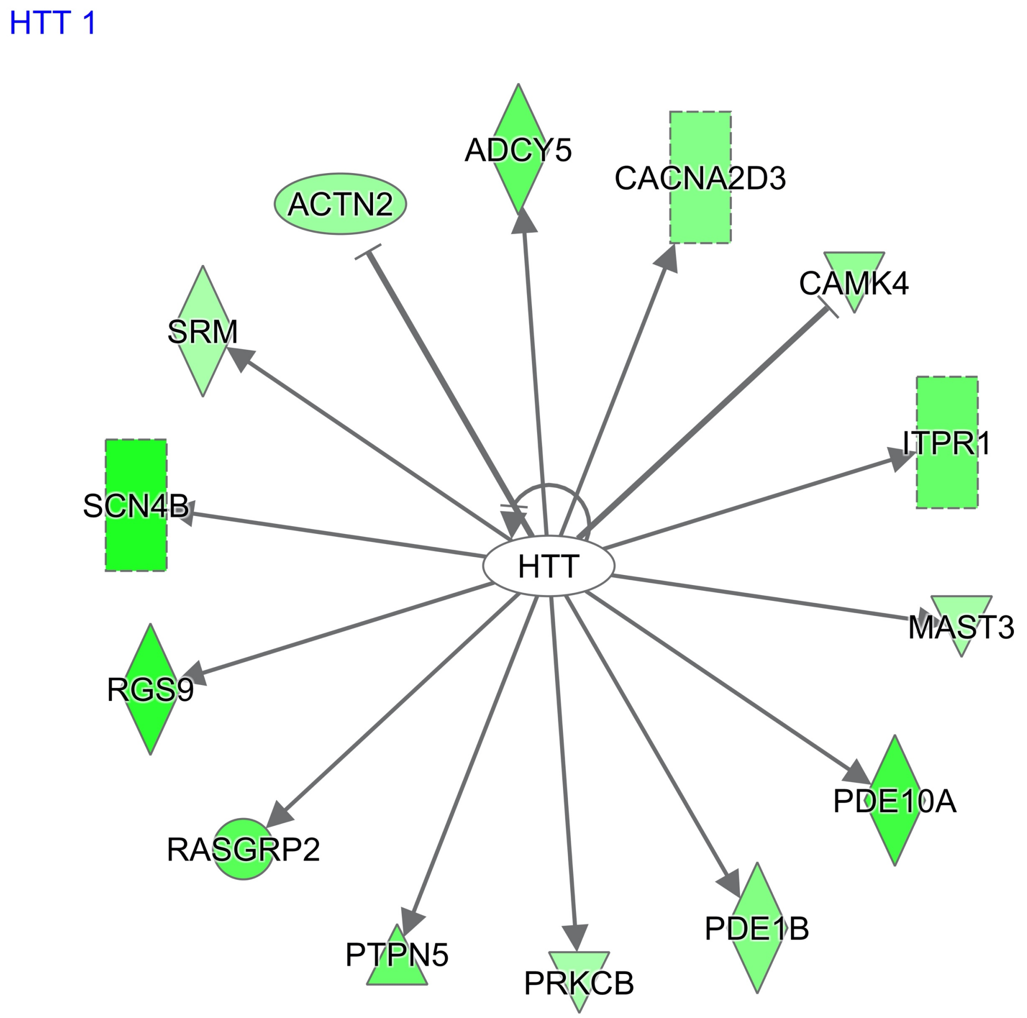

© 2000-2021 QIAGEN. All rights reserved.

Supplementary Figure 6: Upstream regulator in striatal HD proteome
Upstream regulator predicted by IPA for the changes in the proteome in the striatum of HD_SH compared to WT_SH

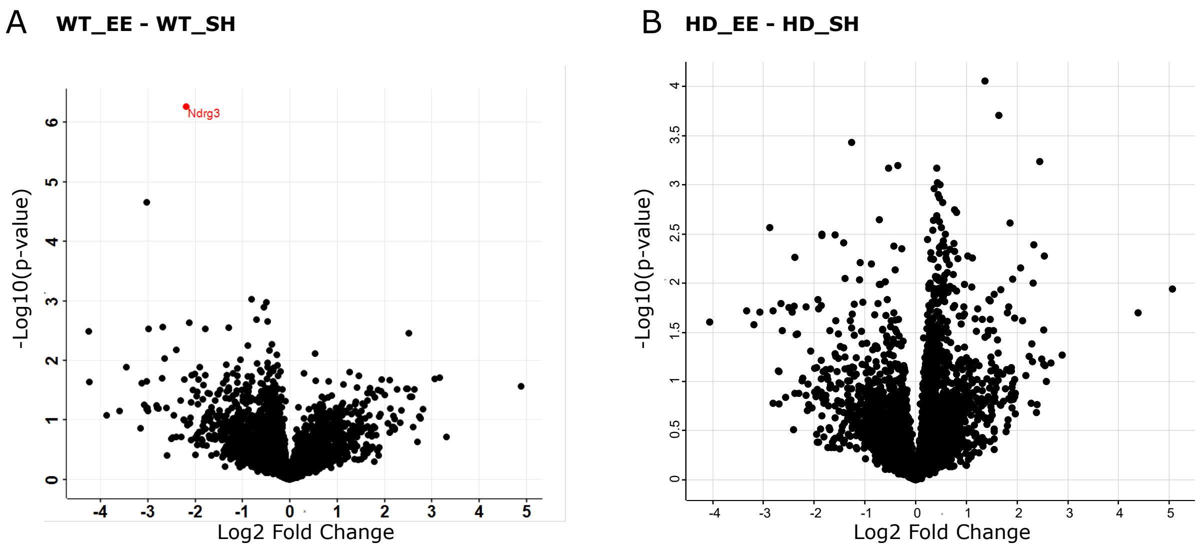

Supplementary Figure 7: Environmental enrichment on striatal phosphoproteome
Volcano plots, indicating statistical significance (-Log p-value) against Log2 fold change for the phosphopeptides between WT in SH and WT in EE (A), and between HD in SH and HD in EE (B). Each dot represents a phosphopeptide. Significant peptides (FDR 5%, FC 1.5) are highlighted in color: red is downregulated in EE, blue is upregulated in EE.

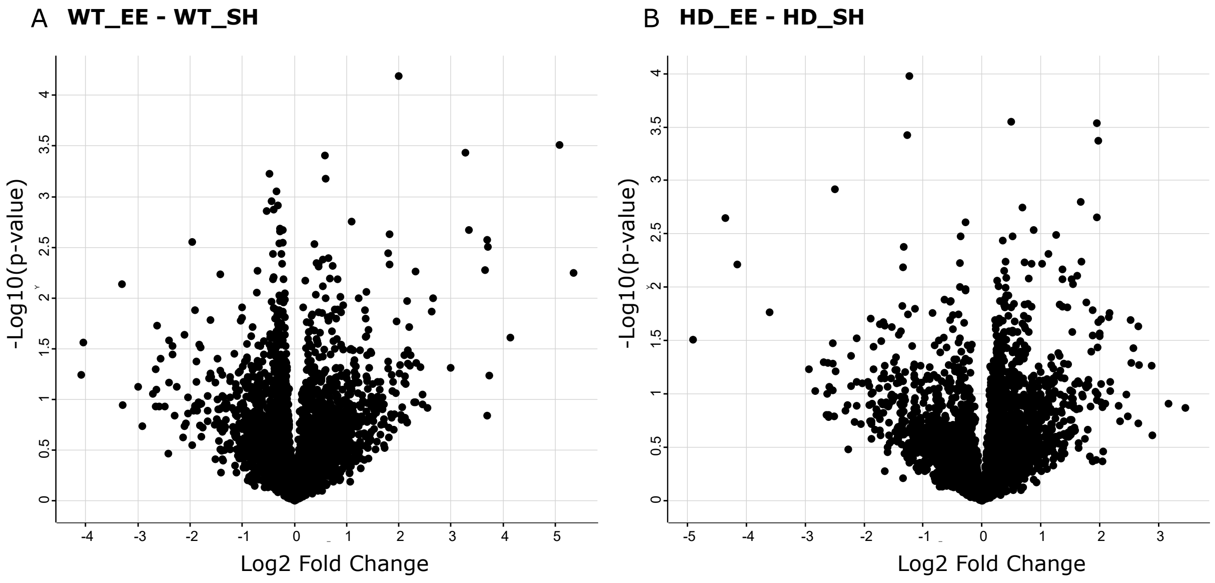

Supplementary Figure 8: Environmental enrichment on hippocampal phosphoproteome
Volcano plots, indicating statistical significance (-Log p-value) against Log2 fold change for the phosphopeptides between WT in SH and WT in EE (A), and between HD in SH and HD in EE (B). Significant peptides are defined as FDR 5% and FC 1.5. Each dot represents a phosphopeptide. No differences were observed between the groups.

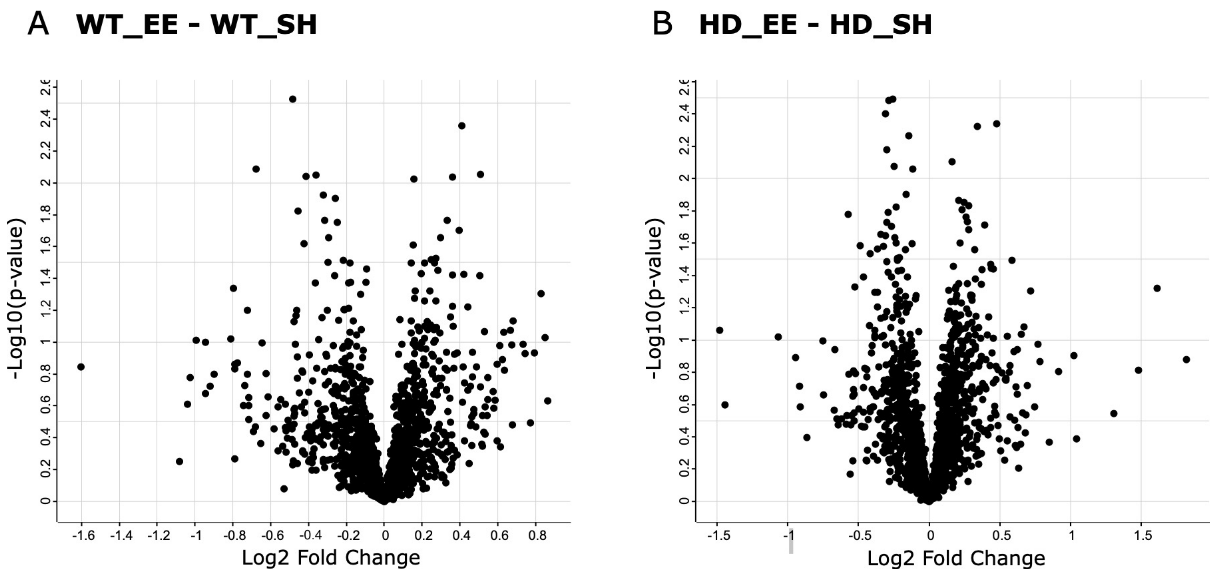

Supplementary Figure 9: Environmental enrichment on striatal proteome

Volcano plots, indicating statistical significance (-Log p-value) against Log2 Fold Change for the significant proteins between WT in SH and WT in EE (A) and between HD in SH and HD in EE (B). Each dot represents a protein. Statistical significance was set at FDR 5% (q-value) and FC 1.5. No differences were observed between the groups.

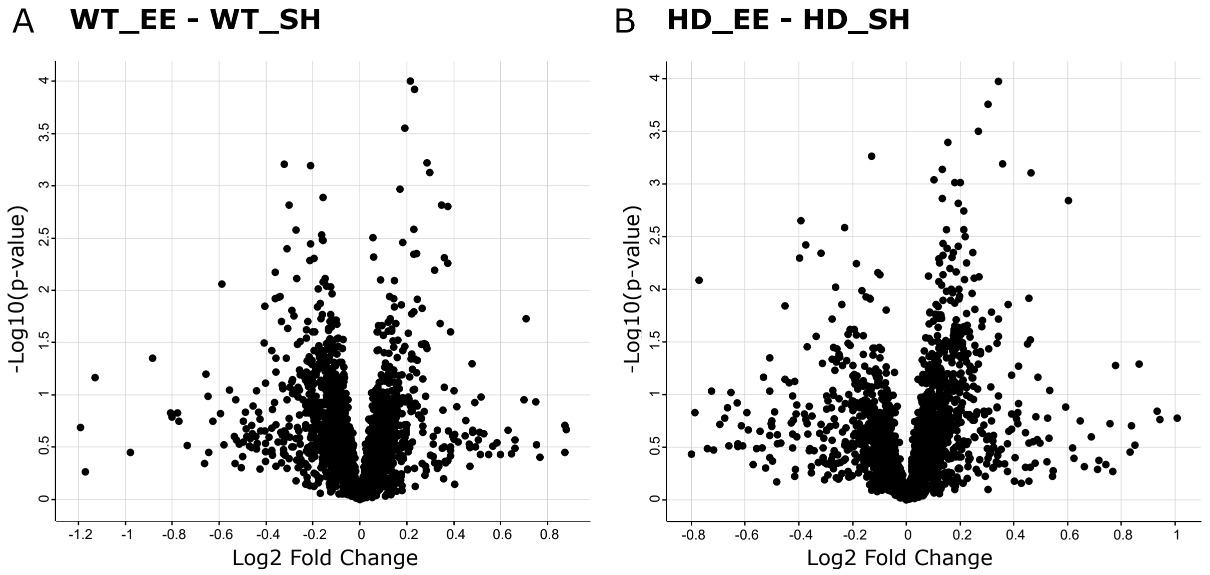

Supplementary Figure 10: Environmental enrichment on hippocampal proteome

Volcano plots, indicating statistical significance (-Log p-value) against Log2 Fold Change for the significant proteins between WT in SH and WT in EE (A) and between HD in SH and HD in EE (B). Each dot represents a protein. Statistical significance was set at FDR 5% (q-value) and FC 1.5. No differences were observed between the groups.

Count	WT_SH	WT_EE	HD_SH	HD_EE
3162	+	+	+	+
241	-	+	+	+
75	-	-	+	+
68	-	+	+	-
36	-	+	-	+
83	+	+	-	+
237	+	+	+	-
85	+	-	+	+
38	+	-	+	-
25	-	-	-	+
24	+	-	-	+
66	+	+	-	-
40	-	-	+	-
38	+	-	-	-
10	-	+	-	-

Supplementary Table 1: Present/Absent Phosphoproteomics analysis in the hippocampus

Table representing the number of phosphopeptide detected in the striatum (count), with the presence of 4 valid values (+) or the absence of 4 valid values (-) in each group.

Count	WT_SH	WT_EE	HD_SH	HD_EE
3444	+	+	+	+
300	+	+	-	+
36	-	+	-	+
23	+	-	-	-
82	+	+	-	-
126	+	+	+	-
91	+	-	+	+
102	-	+	+	+
28	-	-	+	+
44	+	-	-	+
30	-	+	+	-
23	-	-	-	+
8	+	-	+	-
12	-	-	+	-
20	-	+	-	-

Supplementary Table 2: Present/Absent Phosphoproteomics analysis in the hippocampus

Table representing the number of phosphopeptide detected in the hippocampus (count), with the presence of 4 valid values (+) or the absence of 4 valid values (-) in each group.

Count	WT_SH	WT_EE	HD_SH	HD_EE
1769	+	+	+	+
28	-	+	+	+
53	-	-	+	+
64	+	-	+	+
16	+	-	-	-
61	-	-	+	-
22	+	+	+	-
31	-	-	-	+
24	+	-	+	-
18	+	+	-	+
8	+	+	-	-
1	-	+	-	+
8	-	+	-	-
12	+	-	-	+
13	-	+	+	-

Supplementary Table 3: Present/Absent proteomics analysis in the striatum

Table representing the number of proteins detected in the striatum (count), with the presence of 4 valid values (+) or the absence of 4 valid values (-) in each group.

Count	WT_SH	WT_EE	HD_SH	HD_EE
122	+	+	+	-
2597	+	+	+	+
9	-	-	+	+
40	-	+	-	-
16	+	-	+	-
25	-	+	+	-
25	-	+	+	+
40	+	-	-	-
15	-	-	-	+
37	-	-	+	-
39	+	+	-	-
17	+	-	+	+
18	+	+	-	+
11	-	+	-	+
7	+	-	-	+

Supplementary Table 4: Present/Absent proteomics analysis in the hippocampus

Table representing the number of proteins detected in the hippocampus (count), with the presence of 4 valid values (+) or the absence of 4 valid values (-) in each group.
